# Functionality of Root-Associated Bacteria along a Salt Marsh Primary Succession

**DOI:** 10.3389/fmicb.2017.02102

**Published:** 2017-10-30

**Authors:** Miao Wang, Erqin Li, Chen Liu, Alexandre Jousset, Joana F. Salles

**Affiliations:** ^1^Research Group of Microbial Community Ecology, Genomics Research in Ecology and Evolution in Nature, Groningen Institute for Evolutionary Life Sciences, University of Groningen, Groningen, Netherlands; ^2^Plant-Microbe Interactions, Department of Biology, Utrecht University, Utrecht, Netherlands; ^3^Ecology and Biodiversity, Utrecht University, Utrecht, Netherlands

**Keywords:** functionality, plant-associated bacteria, plant selective force, soil type, salt marsh chronosequence

## Abstract

Plant-associated bacteria are known for their high functional trait diversity, from which many are likely to play a role in primary and secondary succession, facilitating plant establishment in suboptimal soils conditions. Here we used an undisturbed salt marsh chronosequence that represents over 100 years of soil development to assess how the functional traits of plant associated bacteria respond to soil type, plant species and plant compartment. We isolated and characterized 808 bacterial colonies from the rhizosphere soil and root endosphere of two salt marsh plants, *Limonium vulgare* and *Artemisia maritima*, along the chronosequence. From these, a set of 59 strains (with unique BOX-PCR patterns, 16S rRNA sequence and unique to one of the treatments) were further screened for their plant growth promoting traits (siderophore production, IAA production, exoprotease production and biofilm formation), traits associated with bacterial fitness (antibiotic and abiotic stress resistance – pH, osmotic and oxidative stress, and salinity) and metabolic potential. An overall view of functional diversity (multivariate analysis) indicated that the distributional pattern of bacterial functional traits was driven by soil type. Samples from the late succession (Stage 105 year) showed the most restricted distribution, harboring strains with relatively low functionalities, whereas the isolates from intermediate stage (35 year) showed a broad functional profiles. However, strains with high trait performance were largely from stage 65 year. Grouping the traits according to category revealed that the functionality of plant endophytes did not vary along the succession, thus being driven by plant rather than soil type. In opposition, the functionality of rhizosphere isolates responded strongly to variations in soil type as observed for antibiotic resistance (*P* = 0.014). Specifically, certain *Pseudomonas* sp. and *Serratia* sp. strains revealed high resistance against abiotic stress and antibiotics and produce more siderophores, confirming the high plant-growth promoting activity of these two genera. Overall, this study contributes to a better understanding of the functional diversity and adaptation of the microbiome at typical salt marsh plant species across soil types. Specifically, soil type was influential only in the rhizosphere but not on the endosphere, indicating a strong plant-driven effect on the functionality of endophytes.

## Introduction

Plant–microbial interactions influence ecosystem functioning through carbon sequestration and nutrient cycling – in natural ecosystems as well as in agricultural systems (Singh et al., 2004; Hussain et al., 2011; Ribeiro and Cardoso, 2012; Hong et al., 2013; Shakya et al., 2013) – thus understanding the drivers of the plant associated microbiome is of great relevance (Berg and Smalla, 2009). Factors such as soil properties and plant species are known to influence the structure and function of microbial communities living in close association with plants, in particular those known as plant growth-promoting rhizobacteria (PGPR; Garbeva et al., 2004a; Jousset et al., 2006; Rasche et al., 2006; Berg and Smalla, 2009). The relative importance of the latter is due to species–specific root exudation patterns, which leads to the enrichment of plant-specific microbial populations in the rhizosphere – the rhizosphere effect (Rovira, 1956; Uren, 2000). For instance, field experiments have shown the influence of plant species on the structure and function of bacterial communities associated with the rhizosphere of three phylogenetically different and economically important crops – strawberry, potato and oilseed rape (Smalla et al., 2001; Berg et al., 2002, 2006; Costa et al., 2006).

The rhizosphere effect varies, however, at different sites because of differences in microbial biogeographical patterns, soil properties and land use (Marschner et al., 2001; Garbeva et al., 2004b; Salles et al., 2006; Lauber et al., 2008), making impossible to disentangle the effects driven by soil type from those related to plant species. By analyzing the phylogenetic distribution of soil bacterial communities along a natural gradient of soils under the influence of the same meta-community and environmental variation, we have recently shown that soil type exerts greater effect on rhizosphere communities than plant, as indicated by the fact that the rhizosphere microbiome followed the changes observed in the bulk soil. However, the endophytic bacterial communities – i.e., those capable of gaining access to the internal root endosphere compartment (Sessitsch et al., 2002; Compant et al., 2005, 2010; Hardoim et al., 2008; Lundberg et al., 2012; Saleem et al., 2015) – were driven by the plant, clustering away from rhizosphere and bulk soil samples and irrespective of soil developmental patterns (Wang et al., 2016). Thus, the intimate relationship between endophytes and plants generated stronger plant selectivity on the phylogenetic distribution of bacterial communities when compared to rhizosphere samples (Hallmann et al., 1997; Rosenblueth and Martínez-Romero, 2006; Schulz and Boyle, 2006).

The phylogenetic information is, however, not always linked to the functionality of the communities (Salles et al., 2012; de Bello et al., 2015) and given the high microbial functional redundancy (Nannipieri et al., 2003; Mendes et al., 2015; Sunagawa et al., 2015), it remains unclear whether similar patterns will arise when functions rather than identity are targeted. The plant microbiome is known to be enriched in functional traits related to root colonization (e.g., transporters), metabolic pathways (e.g., polysaccharide degradation, hydrogen metabolism) and plant growth promoting (PGP) phenotypes (Ofek-Lalzar et al., 2014; Yan et al., 2016). Interestingly, microbial functionality seems dependent on the plant compartment (rhizosphere or endosphere), as shown for phylogenetic information, given that the endosphere microbiome might harbor significantly more metabolic pathways (degradation of multiple aromatic plant metabolites) and PGP phenotypes (production of IAA) than those microbes colonizing the rhizosphere (Timm et al., 2015). It remains unclear however, how soil properties would influence this selection and whether microbial functionality is mostly driven by plant-specific selection or by the soil parameters.

In this study, we aim at exploring the importance of the selective force exerted by the plant in regulating the functionality of plant-associated bacterial species, in different soil types along a salt marsh primary succession chronosequence (Olff et al., 1997; Dini-Andreote et al., 2014, 2015; Wang et al., 2016). We chose *Limonium vulgare* and *Artemisia maritima*, typical perennial salt marsh plants, as our focus species because of their broad distribution along the chronosequence, allowing for a full comparison between plant species, plant compartments and soil types. The bacterial functionality was determined by measuring traits associated with bacterial fitness (antibiotic and abiotic stress resistance – pH, osmotic and oxidative stress, and salinity), metabolic potential determined by growth on different carbon sources, and PGP capacity (siderophore production, IAA production, exoprotease production and biofilm formation). Functionality was assessed by targeting all traits simultaneously (overall functional diversity), specific groups of or individual traits. We hypothesize that the functional diversity of the rhizosphere isolates but not those from root endosphere will increase along succession. Specifically, we expect the functionality of rhizosphere bacterial isolates to change along the soil succession, thus following the increase in the complexity of soil nutrients, organic matter, plant diversity and biomass observed in this system. Conversely, we expect the functionality of those isolates associated with root endosphere to, remain constant, given the plant selectivity and buffering effect previously shown for this system (Dini-Andreote et al., 2014, 2015; Wang et al., 2016).

## Materials and Methods

### Study Site and Sample Collection

Sampling was performed in April in the year 2016. Plant samples were collected at five distinct successional stages in the salt marsh chronosequence. This chronosequence spans more than 100 years of primary succession and is located on the island of Schiermonnikoog (53°30′N, 6°10′E), Netherlands (for detailed information on sampling, see Wang et al., 2016). Samples were collected at locations with successional ages of 5, 15, 35, 65, and 105 years. In this study, we use soil stage as a proxy of soil type, as previous work has revealed that the sedimentation caused by the tidal regime has resulted in modifications on the soil physicochemical conditions along the primary succession, leading to an accumulation of silt and clay particles. In addition, the salinity level also increased over time during succession, due to an accumulative effect. For the details on the establishment of sampling plots and descriptions on the chronosequence verification, see Olff et al. (1997), Dini-Andreote et al. (2014), and Wang et al. (2016). Briefly, triplicated plots within each of the locations were established at the same base of elevation [vertical position relative to mean sea level at the initial elevation gradient on the bare sand flats with a base elevation of 1.16 m ± 2.2 cm (mean ± SE) above Dutch Ordinance Level]. Within each plot, four healthy-looking *L. vulgare* and *A. maritima* of similar sizes with attached soil adhering to the intact roots were collected and processed together generating two composite samples per plot. Thirty composite samples in total were collected (5 stages × 3 plots per stage × 2 plant species). Each sample was placed into a sterile plastic bag, sealed and transported to the laboratory in <24 h. From each composite sample, we separated rhizosphere soil from plant roots (see below).

### Pre-treatment of Rhizosphere Soil and Plant Root Samples

A detailed description for the workflow of the isolation and screening of plant-associated bacteria is given in Supplementary Figure S1A. Rhizosphere soil samples were collected by weighting ten grams of roots with tightly adhering soil particles (about 3 g rhizosphere soil). Root samples were transferred into an Erlenmeyer flask containing 47 mL of sterile 1X Phosphate Buffered Saline (PBS buffer) and shaken for 30 min at 200 rpm at room temperature. An aliquot of 1 mL of the suspension with rhizosphere soil was transferred into sterile 1X PBS buffer and serial dilutions (1/10) were prepared.

Plant roots (about 8 g) were thoroughly washed with running tap water, trimmed to remove adhering soil and dead tissues, followed by surface sterilization, which consisted of immersion in 1.5% NaClO solution for 3 min, followed by 70% ethanol for 3 min and sterile distilled water (3 × 3 min). The surface-sterilized root samples (5 g) were diced with a sterile scalpel and immersed into 45 mL of 0.9% NaCl solution. After shaking incubation for 1 h at 28°C, the suspension with root pieces was shaken using a horizontal vortex instrument (4 × 1 min, 30 s in-between). An aliquot of 1 mL of the suspension containing the released plant endophytes was transferred into sterile 1X PBS buffer and serial dilutions (1/10) were prepared. Sterility checks were performed by tissue-blotting surface-sterilized root samples on R2A plates at 28°C for 2–7 days. Only samples without bacterial growth were considered successfully sterilized and used further.

### Bacterial Isolation and Identification from Rhizosphere Soil and Plant Root Samples

R2A medium, as a non-selective medium recommended for the examination of total heterotrophic bacteria in soil (Ellis et al., 2003), was used to culture the heterotrophic population. Aliquots of 0.1 mL of the 10^-1^ to 10^-3^ dilutions from rhizosphere soil and root samples were spread on R2A medium plates and incubated for 2 days at 25°C, after which we determined the number of colony forming units (CFU). A maximum of 32 bacterial colonies with unique morphologies per replicate were purified using a streak-plate procedure (see Supplementary Table S1 for total numbers), transferred onto new R2A medium plates and further used as templates for BOX-PCR – a DNA-based typing method that differentiate bacterial species at strain level by simultaneously screening DNA regions scattered in the bacterial genome (Brusetti et al., 2008). To improve cell lysis, the colonies were first inoculated into 50 μL of NaOH solution (0.05 M) and then lysed at 95°C for 15 min in the PCR machine. BOX-PCR was performed by using the BOX-A1R primer (5′-CTACGGCAAGGCGACGCTGACG-3′) (Versalovic et al., 1994). Twenty μL PCR reactions were performed using 0.32 μL 5 U μL^-1^ Taq DNA Polymerase, 4 μL of 5X Gitschier Buffer [83 mM (NH_4_)_2_SO_4_, 335 mM Tris-HCl (pH 8.8), 32.5 mM MgCl_2_, 325 mM EDTA (pH 8.8), 1% commercial stock of β-mercaptoethanol, ddH2O], 2 μL 100% DMSO, 1 μL 25 mM of each dNTP in a mixture, 0.32 μL 20 mg mL^-1^ bovine serum albumin (BSA) (Roche Diagnostics GmbH, Mannheim, Germany), and 0.8 μL of 10 μM BOX-A1R primer and 1 μL of the lysed cell solution. The thermal cycler protocol was 95°C for 3 min, 35 cycles of 94°C for 4 s, 92°C for 30 s, 50°C for 60 s, 65°C for 8 min and a final 16 min extension at 65°C. BOX-PCR profiles were visualized by separation on 2% agarose gel and staining with ethidium bromide. Images of the gels were visualized and documented under UV light with Image Master VDS system (Amersham Biosciences, United Kingdom). For the details of the bacterial isolates showing unique BOX-PCR profiles, see Supplementary Tables S1 and S2. Culture stocks for individual isolate were stored in 25% glycerol at -80°C.

### Molecular Characterization of Bacterial Isolates

A total of 159 bacterial cultures with unique BOX-PCR patterns (Supplementary Figure S1 and Table S2) were subjected to total DNA extraction using the MoBio UltraClean Microbial DNA Isolation Kit (MoBio Laboratories, Carlsbad, CA, United States). We followed the instruction manual, except for heating the preparations at 65°C for 10 min with occasional bump vortexing for a few seconds every 2–3 min. The amount of DNA in each sample was quantified using a NanoDrop ND-1000 spectrophotometer (NanoDrop Technologies). All DNA samples were standardized to the equal concentration of 5 ng μL^-1^ for further analyses.

16S rRNA gene amplification was performed by using the bacterial-specific primers, B8F (5′-AGAGTTTGATCMTGGCTCAG-3′) (Edwards et al., 1989) and U1406R (5′-ACGGGCGGTGTGTRC-3′) (Lane, 1991). Fifty μL PCR reactions were performed with 0.2 μL 5U μL^-1^ FastStart High Fidelity (FSHF) Taq DNA Polymerase, 5 μL 10X FSHF Reaction buffer without MgCl_2_, 0.8 μL 50 mM MgCl_2_ stock solution, 1 μL 10 mM PCR nucleotide mix, 0.5 μL 20 mg mL^-1^ BSA (Roche Diagnostics GmbH, Mannheim, Germany), and 1 μL each of 10 μM primer and 5 ng DNA template. The thermal cycler protocol was 95°C for 5 min, 35 cycles of 95°C for 60 s, 52°C for 30 s, 72°C for 2 min and a final 7 min extension at 72°C. Amplicons were run with 1% (w/v) agarose gel to check the band size, and then sequenced at LGC Genomics GmbH (Berlin, Germany) on an Applied Biosystems^®^ 3730XL DNA analyzer. The 16S rRNA gene sequences of bacterial isolates were compared with the reference sequences available in the Nucleotide Database of the National Center for Biotechnology Information (NCBI) using the basic local alignment search tool (Nucleotide BLAST)^1^. The accession numbers of the 68 unique strains identified at species level by the 16S rRNA gene sequences are MF664115 to MF664179 and MF677865 to MF677867 (Supplementary Table S3). Phylogenetic and molecular evolutionary analyses with the 16S rRNA gene sequences of bacterial isolates were conducted by using software MEGA 7.0 for bigger datasets (Kumar et al., 2016). The sequences were aligned by using the CLUSTALW (Thompson et al., 1997). Tree constructions were performed using the Maximum Likelihood [ML] method (Cavalli-Sforza and Edwards, 1967; Felsenstein, 1981, 1993). The robustness of the phylogenetic tree was confirmed by using 1000 bootstrap replications. Additionally, *Chlorella vulgaris* C-27 and *Planctomycetes bacterium* AS92 were included as the outgroup, and the validation of the phylogenetic neighbors was carried out by adding the 16S rRNA sequences of type strains obtained from the SILVA rRNA database project (Quast et al., 2013; Yilmaz et al., 2014). Phylogenetic tree was visualized and exported using the web-based tool Interactive Tree Of Life (iTol) (Letunic and Bork, 2011).

### Biochemical Assays for Functional Traits by Using Microtiter Plate

#### Plant Growth Promoting Traits

A detailed description of the functional traits screening for the plant-associated bacteria is given in Supplementary Figure S1B. For screening the PGP traits, bacterial culture supernatant was first collected. That was achieved by growing the individual isolates in 96-well microtiter plates containing Luria-Bertani (LB) medium. Plates were incubated at 25°C for 2 days, when cells reached the stationary phase (as determined by measuring absorbance at 600 nm). Plates were then centrifuged (2800 rpm for 30 min) and the supernatant was filtered through a 96-well microtiter filter plate. For each microtiter plate, negative controls were included by adding LB medium without bacterial culture together with other reagents.

The ability to produce siderophore was qualitatively determined by using the modified chrome azurol S (CAS) solution according to Alexander and Zuberer (1991). The standard curve for the determination of siderophore concentration was prepared by diluting 1 mM FeCl_3_ into 100 mL of the CAS solution, with the final concentrations of 0, 0.005, 0.01, 0.015, 0.03 mM FeCl_3_ in CAS solution. The concentration of siderophore in the culture filtrates was screened by mixing 50 μL bacterial supernatant with 100 μL CAS solution containing 0.015 mM FeCl_3_ (modified CAS solution) in a 96-well microtiter plate. The mixtures and the standard curve were equilibrated for 3–4 h and the amount of consumed FeCl_3_ in the mixture – considered as the proxy of siderophore production – was subsequently determined by measuring absorbance at 630 nm with an automated microplate reader (Bio-Tek Instruments).

IAA production was determined colorimetrically (Gordon and Weber, 1951; Glickmann et al., 1995). Briefly, 10 μL of bacterial supernatant was mixed with 15 μL of Salkowski-reagent (1.2% FeCl_3_ in 37% H_2_SO_4_) (ratio bacterial supernatant to Salkowski-reagent 1:1.5) in a 96-well microtiter plate, and the mixture was incubated overnight at room temperature. The colorations from yellow to purple indicating IAA production were then measured at 535 nm. The amount of IAA presented in the supernatant was determined by comparing the observed data to a standard curve generated by diluting synthetic IAA to concentrations between 10^-6^ and 10^-4^.

Exoprotease activity was quantitatively measured by azocasein colorimetry method, modified from Smeltzer et al. (1992). Briefly, 15 μL culture supernatant was mixed with 25 μL of 2% azocasein in a 96-well microtiter plate, and the mixture was incubated at 40°C for 24 h. Then 125 μL of 10% Tricholoracetic acid (TCA) was added the mixture. After centrifugation (5 k rpm for 30 min), 100 μL of the supernatant was neutralized with 100 μL of 1 M NaOH in a new microtiter plate, followed by the colorimetric measurement at 440 nm.

Biofilm formation was screened using the microtiter plate biofilm assay modified from O’Toole and Kolter (1998). Briefly, bacterial strains were inoculated into 96-well microtiter plates containing 200 μL of LB medium and incubated at 25°C for 2 days, reaching the stationary phase. Subsequently, 150 μL bacterial culture were transferred to new 96-well microtiter plates, covered with a lid with pegs (TSP, NUNC, Roskilde, Denmark) and the plates were incubated for 24 h at room temperature to allow biofilm formation. Next, the pegs with biofilm were washed in a tray with 1X PBS (five times) to remove bacterial cells, and stained for 20 min in 160 μL of 1% crystal violet solution. The peg was then transferred to a microtiter plate with 200 μL 96% ethanol and incubated for 20 min. The crystal violet concentration in ethanol representing biofilm formation was measured by absorbance at 590 nm.

#### Bacterial Fitness Traits

For screening the bacterial fitness traits, bacterial cultures growing to the stationary phase were used (see PGP traits for details). For each microtiter plate, negative controls were included by adding LB medium without bacterial culture together with other reagents.

Biotic stress resistance was measured by determining the resistance to the antibiotics of streptomycin derived from *Streptomyces* (bacteria) and the fungal secreted penicillin. Specifically, 2 μL of bacterial cells were transferred to individual wells on a 96-well microtiter plate containing 160 μL LB medium and the antibiotics, at final concentration of 1 μg/mL. Plates were incubated for 2 days at 25°C, when bacterial growth was measured at the wavelength of 600 nm.

Abiotic stress resistance was measured by screening the resistance to different pH values (pH = 5, 6, 8, 9), salinity (final concentration of NaCl, w/v 7 and 10%), oxidative (final concentration of H_2_O_2_, 0.00025 and 0.0005%) and osmotic (15% Polyethylene glycol Mn6000 PEG) stress. Similarly to the previous tests, 2 μL of bacterial cells were transferred to individual wells on a 96-well microtiter plate containing 160 μL LB medium and the respective reagent. Incubation period and plate reading was performed as for biotic stress.

### Metabolic Potential Determined by Growth on Different Carbon Sources

To test the effect of different types of carbon sources on the bacterial growth, 14 carbon sources including Alanine, Arabinose, Butyrolactam (2-Pyrrolidone), Fructose, Galactose, Glucose, Glycerol, Glycine, Lactic Acid, Putrescine, Serine, Succin Acid, Threonine, Valine were used and incorporated individually into modified OSG medium (minimal medium containing all salts required for bacterial growth) (Schnider-Keel et al., 2000) (15 μL carbon source into 135 μL modified OSG medium) at the final concentration of 0.05% (w/v). After inoculation of 5 μL bacterial culture into the 150 μL modified OSG medium containing corresponding carbon source onto a 96-well microtiter plate, followed by incubation for 2 days at 25°C, growth was measured by absorbance at 600 nm. For each microtiter plate, negative controls were included by adding LB medium without bacterial culture together with the other reagents.

### Data Analysis

The statistical analyses in this study were performed in R environment^2^ (R Core Team, 2013). CFU values were log transformed before statistical analysis. Significant differences in log (CFU) across plant compartments, plant species, and soil types were identified using three-way analysis of variance (ANOVA) (function *aov*), followed by multiple comparisons (function *TukeyHSD*) in multcomp package (Hothorn et al., 2008). Prior to ANOVA analyses, normality of the dataset was tested by using base function *sapply()* modified by adding the function *mystats* by Kabacoff (2015), while homogeneity of variances was verified by using the function *fligner()* in R.

The data associated with the functional traits (biochemical assays) were subjected to a range of normalizations. First, the absorbance values obtained for bacterial fitness (abiotic and biotic resistance) and metabolic potential (growth in 14 carbon sources) were normalized by the absorbance of the bacterial growth in LB medium without other reagents at the stationary phase. Traits associated with bacterial fitness were further weighted to account for the relative abundance of each strain in the original samples. This was achieved by multiplying the absorbance data by the relative abundance of each strain, i.e., the ratio of the number of colonies identified as a specific strain to the total number of colonies obtained from the corresponding treatment. For the traits associated with the metabolic potential, the normalized data were further standardized by the maximum absorbance value observed across all carbon sources and used to calculate the niche breadth for each bacterial isolate, by summing all 14 values according to Salles et al. (2009). Regarding the traits associated with plant growth promotion, which are based on bacterial supernatant, the absorbance values were only weighted to account for the relative abundance of each strain in the original samples, as described above.

In order to show the variation of functionality along the chronosequence, the variation of individual functional traits along the chronosequence were simulated using polynominal regression performed in polynom package (Venables et al., 2016) and plotted in ggplot2 package (Wickham, 2009), and only the significant regressions were shown. The polynomial regression was used to verify the distribution of traits along the chronosequence containing five successional stages, as previously done for data referring to plant diversity and biomass, bacterial diversity and microbial activities (Schrama et al., 2013; Dini-Andreote et al., 2014, 2015; Wang et al., 2016; Salles et al., 2017). To test for correlations between bacterial growth under pH and salinity stress and the variations of soil pH and salinity along the succession, we applied Spearman’s rank-based correlational analysis. The distribution of functional traits performed by isolates from different successional stages and plant compartments was performed by principal components analysis (PCA), using the built-in R function *prcomp()*. Values of the individual functional traits were log transformed before PCA. The visualization of the PCA results was conducted by using the function *ggbiplot()* in the packages ggbiplot (Vincent, 2011).

To further verify the overall patterns of functional diversity, we generated functional diversity measure encompassing all the quantified traits, by modifications from Laliberté and Legendre (2010). A matrix containing all normalized data on individual trait for each species was used to calculate the pairwise distance between species based on Bray Curtis distance. We then calculated the average pairwise distance for each treatment (plant compartment, plant species and soil type) by pooling the treatment-specific species together. The pairwise comparisons for the overall functional diversity between two stages were tested by using *Post hoc* test (function *posthoc.kruskal.nemenyi.test*) in PMCMR package (Pohlert, 2014) after Kruskal–Wallis test.

The strength of each functional trait associated with the bacterial isolates from different plant compartments, plant species and soil types were visualized by using heatmap (function *aheatmap*) in NMF package (Gaujoux, 2015; Seoighe, 2015). Prior to analysis, the normalized absorbance values were further standardized by using *Z*-scores. Pie charts were used to summarize the proportion of bacterial isolates from one of the treatments.

## Results

### Isolation, Screening and Characterization of Plant-Associated Bacteria in Rhizosphere and Root Endosphere

**FIGURE 1:**
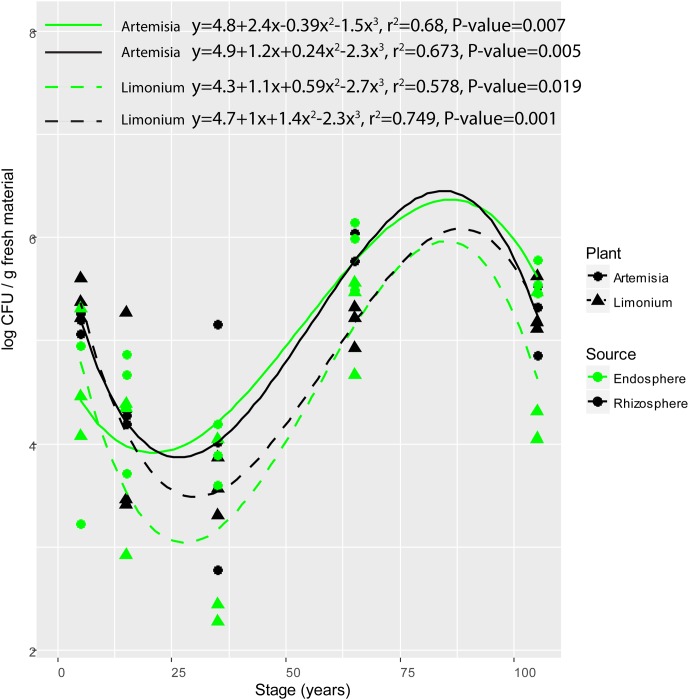
Variation of colony forming units (CFU) of root-associated bacterial isolates along the chronosequence. For plant species, solid and dashed line represent *A. maritima* and *L. vulgare*, respectively. For plant compartments, black refer to rhizosphere isolates whereas green represent those obtained from the endosphere.

The population counts on R2A agar plates of bacterial isolates from the rhizosphere soil and root endosphere of *L. vulgare* and *A. maritima* along the chronosequence were significantly influenced by soil type (*F* = 10.820, *P* = 0.002). Significant differences in population counts were respectively observed between middle (15- and 35-year stages) and late (65- and 105-year stages) successional phases for either rhizosphere or endosphere from both plants (Tukey’s HSD test, for *L. vulgare*, rhizosphere, *P* = 0.012, endosphere, *P* = 0.008; for *A. maritima*, rhizosphere, *P* = 0.013, endosphere, *P* = 0.001). In addition, significant polynomial variations were found for either compartment from both plant species (**Figure 1**), respectively decreasing from the initial (5-year stage) to middle phase followed by an increase toward the late phase along the chronosequence.

In order to foster variation among isolates, up to 30 colonies differing in morphology were recovered from each treatment, generating a total of 808 colonies. These were characterized by genotypic characterization using BOX-PCR, which targets repeated regions across the bacterial genome, therefore depicting differences at strain level (thus beyond 16S rRNA gene identification, Cho and Tiedje, 2000). The BOX-PCR analyses indicated that these 808 colonies could be assigned to 159 bacterial genotypes (strains) with unique BOX-PCR patterns (Supplementary Figure S1 and Table S1), which were further identified at species level by sequencing of the 16S rRNA gene, revealing a set of 68 unique bacterial species (Supplementary Table S2). From those, 59 species were unique to individual treatments and further functionally characterized using biochemical assays targeting a range of traits. The remaining 9 strains were observed in more than one plant compartment or plant species (*Hafnia psychrotolerans strain CSE_16*, *Pseudomonas sp. CanL-3*, *Pseudomonas sp. 332*, *Pseudomonas sp. UT 6-06*, *Pseudomonas sp. ARCTIC-P37*, *Pseudomonas sp. JY-Q*, *Pseudomonas sp. 46 (2016)*, *Pseudomonas fluorescens strain FW300-N2E2*, and *Serratia fonticola strain 51*) and therefore not included to avoid biases in the analyses (Supplementary Figure S2).

According to phylogenetic analyses, the 59 unique strains were affiliated with six taxonomic groups (Supplementary Figure S3). Gammaproteobacteria was the predominant class with 45 unique strains included, from which 33.3% of the strains were isolated from the 65-year stage while the remaining ones were almost evenly distributed among other stages. Moreover, the strains belonging to Gammaproteobacteria were equally isolated from either plant species or plant compartment (Supplementary Figure S4). Actinobacteria was the second dominant phylum including 6 unique strains, from which 3 strains were obtained from 5-year stage. Among these Actinobacteria isolates, 66.7% were from *A. maritima*, among which 75% were from rhizosphere. The strains affiliated with Bacilli (two isolates) and Bacteroidetes (four isolates) were all isolated from the rhizosphere of *A. maritima*, whereas the single Alpha- and Beta-proteobacteria isolates were from the endosphere of *L. vulgare*. Specifically, the strains belonging to Bacteroidetes, Alpha- and Beta-proteobacteria were only isolated from 5-year stage. Thus, the diversity of bacterial isolates was highest at 5-year stage including five taxonomic groups (16 unique strains), among which Gammaproteobacteria accounted for almost half of the total amount of strains (Supplementary Figure S5). Whereas the lowest taxonomic diversity was observed at 65-year stage, where 14 out of 15 isolated strains were affiliated with Gammaproteobacteria.

### Dynamics of Biochemical Properties of Plant-Associated Bacteria in Response to the Chronosequence

**FIGURE 2:**
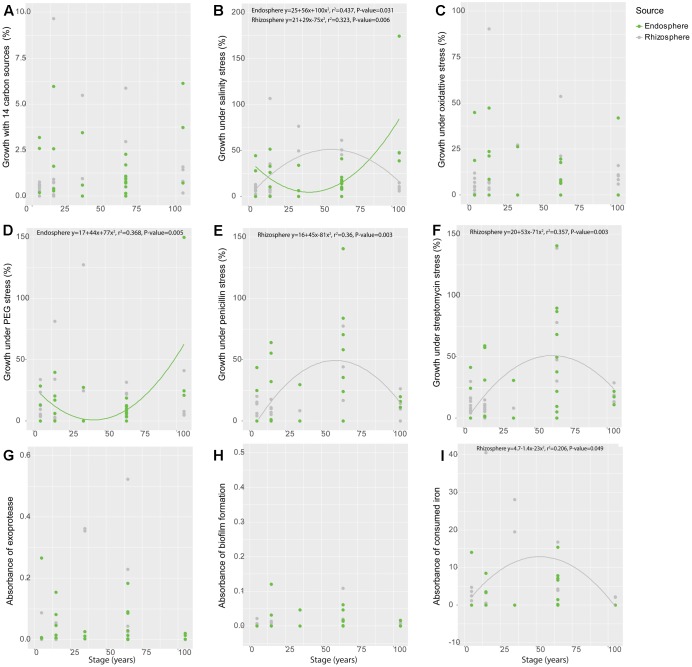
Variation of functional traits of bacterial isolates from rhizosphere and endosphere along the chronosequence. **(A)** Metabolic potential, **(B)** Salinity stress resistance, **(C)** Oxidative stress resistance, **(D)** Osmotic stress resistance, **(E)** Penicillin resistance, **(F)** Streptomycin resistance, **(G)** Exoprotease production, **(H)** Biofilm production and **(I)** Siderophore production. Gray color refer to rhizosphere isolates whereas green represent those obtained from the endosphere.

When analyzed along time – as represented by the different soil stages along the primary succession – some of the individual functional traits showed significant patterns along the chronosequence. Specifically, isolates obtained from the rhizosphere showed a hump-shaped distribution for salinity stress resistance (*r*^2^ = 0.323, *P* = 0.006) and siderophore production (*r*^2^ = 0.206, *P* = 0.049), peaking at the 35-year stage (**Figures 2B,I**). The salinity stress resistance strongly correlated with soil salinity, whose variation progressively increases along the succession (Wang et al., 2016) (spearman correlation, *P* = 0.005; polynomial regression, *r*^2^ = 0.219, *P* = 0.042) (Supplementary Figure S6). Similarly, hump-shaped tendencies were found for antibiotics resistance (penicillin: *r*^2^ = 0.360, *P* = 0.003; streptomycin: *r*^2^ = 0.357, *P* = 0.003), but those traits peaked later in the succession, at the 65-year stage (**Figures 2E,F**). Regarding the isolates obtained from the endosphere, we only found significant patterns for resistance to salinity (*r*^2^ = 0.437, *P* = 0.031) and osmotic stress (*r*^2^ = 0.368, *P* = 0.005) along the chronosequence (**Figures 2B,D**), showing a reverse hump-shaped pattern with a slight decrease from the initial stage to 65-year stage followed by a sharp increase toward 105-year stage, while other traits were relatively constant across the succession (**Figures 2A,C,E–I**). For both rhizosphere and endosphere, no significant trend of bacterial growth under different pH was observed (Supplementary Figures S7, S8).

### Variation in Functional Diversity along the Chronosequence

**FIGURE 3:**
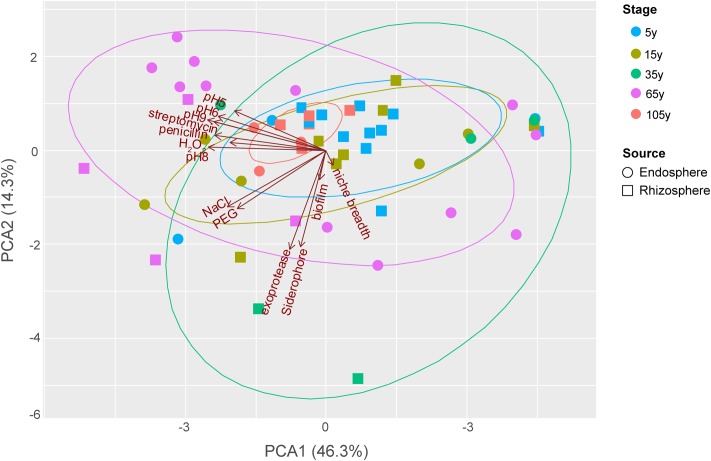
Principal components analysis (PCA) biplot of the functionalities of bacterial isolates from different plant compartments along the chronosequence. For successional stages, blue, light beige, green, purplish red and orange color represent 5, 15, 35, 65 and 105-year stage, respectively. For plant compartments, square refer to rhizosphere isolates whereas circle represent those obtained from the endosphere.

The functional trait patterns obtained from the bacterial isolates from different plant compartments across the succession were represented using multivariate analyses (PCA, **Figure 3**). PC1 and PC2 accounted for 60.67% of the total variance among all traits tested in this study, largely reflecting the functionalities of bacterial isolates. PC1 was mostly represented by antibiotic resistance, and growth under different pH (especially pH = 6, 8, 9). PC2 was mainly represented by production of exoprotease and siderophore. In addition, resistance to salinity stress is associated with a PC3 (not shown), given the 45° angle with both PC1 and PC2.

The distributional pattern of functional traits of bacterial isolates from each stage was unique. Stage 105 year showed the most restricted distribution among others as most of these strains performed relatively low functionalities (**Figure 3**), except for two endosphere isolates showing relatively high growth level under different pH as well as resistance to salinity and osmotic stress. Conversely, the isolates from stage 35 year showed a broad functionality, given their great dispersion in the PCA. Among these small number of strains with high functional profiles, one rhizosphere isolates showed high production of exoprotease and siderophore, whereas one endosphere isolate was found to show high resistance to antibiotics and high growth level under different pH. Similarly, the dispersion level of isolates from 65-year stage was also high. However, a large number of these isolates, which are mostly endophytes, showed high growth level under different pH, while two rhizosphere isolates were found to perform high resistance to salinity and osmotic stress as well as the production of exoprotease and siderophore. The dispersion level of isolates from 5- and 15-year stages was moderate compared to others. In addition, most of the isolates these two stages showed relatively low functional profiles, except for one endosphere isolate with high antibiotic resistance and two other endosphere isolates with high resistance to salinity and osmotic stress from 15-year stage.

**FIGURE 4:**
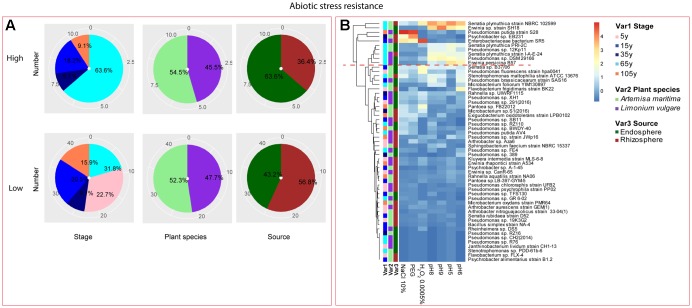
Distribution of abiotic stress resistance of root-associated bacterial strains. **(A)** Distribution of isolates from different treatments according to high and low activities in abiotic stress resistance. **(B)** Heatmap profiles for abiotic stress resistance of all isolates. The annotation of treatments – successional stages, plant species and plant compartments were respectively referred as Var1, Var2 and Var3. For successional stages, pink, blue, navy, cyan and orange color represent 5, 15, 35, 65, and 105-year stage, respectively. For plant species, purple refers to *L. vulgare* and green to *A. maritima*. For plant compartments, red refer to rhizosphere and green to endosphere isolates. The blue-red gradient bar represents the normalized absorbance values (*Z*-scores). Only the integer scaled values (0–4) were shown alongside the color bar. The red dashed line separates the high and low bacterial functional profiles, which were determined by the cluster assignment based on the average Euclidean distance among the bacterial strains.

**FIGURE 5:**
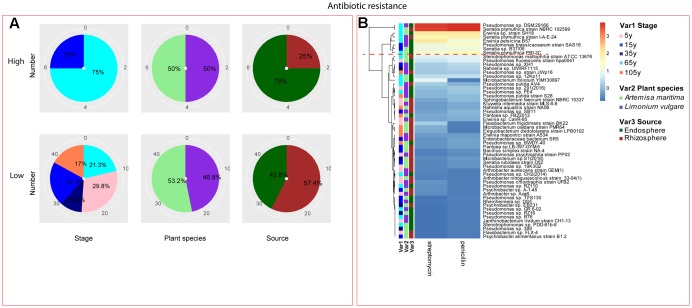
Distribution of antibiotic resistance of root-associated bacterial strains. **(A)** Distribution of isolates from different treatments according to high and low activities in antibiotic resistance. **(B)** Heatmap profiles for antibiotic resistance of all isolates. The annotation of treatments – successional stages, plant species and plant compartments and corresponding colors are the same with those in **Figure 4**. The blue-red gradient bar represents the normalized absorbance values (*Z*-scores). Only the integer scaled values (0–3) were shown alongside the color bar. The red dashed line separates the high and low bacterial functional profiles, which were determined by the cluster assignment based on the average Euclidean distance among the bacterial strains.

**FIGURE 6:**
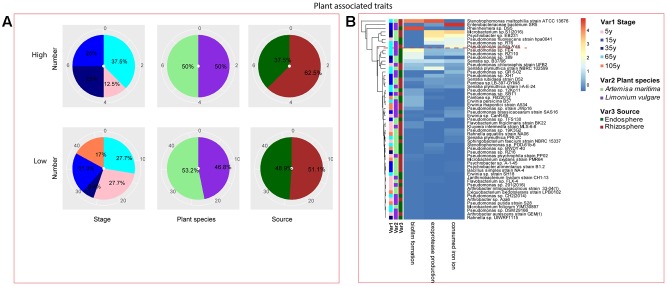
Distribution of plant growth promoting traits of root-associated bacterial strains. **(A)** Distribution of isolates from different treatments according to high and low growth profiles in plant growth promoting traits (biofilm formation, production of exoprotease and siderophore). **(B)** Heatmap profiles for plant growth promoting traits of all isolates. The annotation of treatments – successional stages, plant species and plant compartments and corresponding colors are the same with those in **Figure 4**. The blue-red gradient bar represents the normalized absorbance values (*Z*-scores). Only the integer scaled values (0–4) were shown alongside the color bar. The red dashed line separates the high and low bacterial functional profiles, which were determined by the cluster assignment based on the average Euclidean distance among the bacterial strains.

**FIGURE 7:**
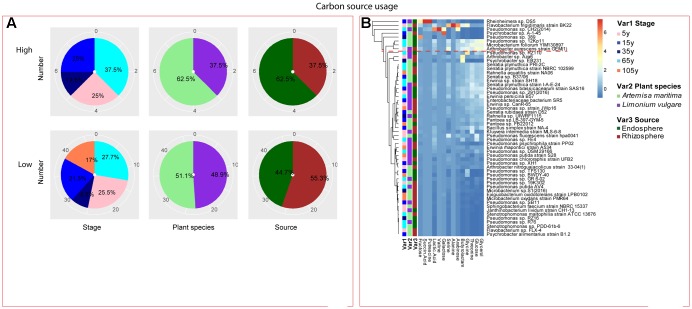
Distribution of metabolic potential of root-associated bacterial strains. **(A)** Distribution of isolates from different treatments according to high and low growth profiles in metabolic potential. **(B)** Heatmap profiles for metabolic potential of all isolates. The annotation of treatments - successional stages, plant species and plant compartments and corresponding colors are the same with those in **Figure 4**. The blue-red gradient bar represents the normalized absorbance values (*Z*-scores). Only the integer scaled values (0–6) were shown alongside the color bar. The red dashed line separates the high and low bacterial functional profiles, which were determined by the cluster assignment based on the average Euclidean distance among the bacterial strains.

Among the isolates showing high biochemical properties, which were mostly from 65-year stage, *Pseudomonas sp. DSM29166*, *Serratia plymuthica strain NBRC102599*, *Serratia plymuthica PRI-2C*, *Serratia plymuthica strain I-A-E-24*, *Erwinia sp. strain SH18*, and *Erwinia persicina B57* showed both resistance to abiotic stress and antibiotics (**Figures 4**, **5**). Strains showing high functional profile in plant associated traits were also largely from 65-year stage (37.5%) compared to other stages, including *Pseudomonas sp. R76*, *Pseudomonas putida AV4* and *Stenotrophomonas maltophilia strain ATCC 13676* (**Figure 6**). Similarly, a larger proportion of the isolates showing high metabolic potential were from 65-year stage (37.5%) compared to others, including *Pseudomonas sp. CH2(2014)*, *Pseudomonas sp. 12Kp11* and *Microbacterium foliorum YIM130897* (**Figure 7**).

To further verify the patterns of functional diversity, we generated functional diversity measures encompassing all the traits measured, allowing us to depict functional similarity among species along a continuum of soil formation as a whole (total functional diversity) or per category of function (specific functional diversity): traits associated with bacterial fitness (antibiotic and abiotic stress resistance – pH, osmotic and oxidative stress, and salinity), metabolic potential determined by growth on different carbon sources, and PGP capacity (siderophore production, exoprotease production and biofilm formation).

Analyzes of variance indicated soil type exerted significant influence on total functional diversity (*P* = 0.035), but not on that for bacterial isolates from either plant compartment (*P* > 0.05) (**Table 1**). In addition, the total functional diversity at the 35-year stage was higher compared with the initial and late stages, confirming the broad distribution observed in the PCA. When grouping the functional traits according to soil stages, we observed that soil type influenced the functional trait diversity associated with antibiotics resistance (*P* = 0.014) in rhizosphere with a significant enrichment at the 35-year stage, whereas for endophytes, none of the diversity of functional traits was affected by stage (*P* > 0.05) (**Table 1**). Plant species exerted significant effect on the functional diversity related to resistance against abiotic stress, metabolic potential and PGP capacity (respectively, *P* = 0.004, *P* = 0.001, *P* = 0.049) (**Table 2**). Moreover, the total functional diversity of isolates obtained from *L. vulgare* followed the development of succession, peaking at the 15-year stage (**Table 1**).

**Table 1:** Functional diversity indices for different successional stages.

Traits	Source	Stage	Functional diversity	*P*-value (K-W test)
Total	Both rhizosphere and endosphere	5 year	0.69 ± 0.23	0.035^∗^
		15 year	0.71 ± 0.31	
		35 year	0.88 ± 0.17	
		65 year	0.64 ± 0.32	
		105 year	0.61 ± 0.25	
Total	Rhizosphere	5 year	0.71 ± 0.24	0.504
		15 year	0.73 ± 0.34	
		35 year	0.64 ± 0.00	
		65 year	0.61 ± 0.21	
		105 year	0.63 ± 0.28	
	Endosphere	5 year	0.67 ± 0.32	0.520
		15 year	0.73 ± 0.27	
		35 year	0.98 ± 0.03	
		65 year	0.65 ± 0.33	
		105 year	0.63 ± 0.17	
Total	*Artemisia maritima*	5 year	0.85 ± 0.20	0.141
		15 year	0.72 ± 0.34	
		35 year	0.93 ± 0.13	
		65 year	0.56 ± 0.36	
		105 year	0.73 ± 0.17	
	*Limonium vulgare*	5 year	0.56 ± 0.20	0.02^∗^
		15 year	0.75 ± 0.24	
		35 year	N.A.	
		65 year	0.69 ± 0.28	
		105 year	0.33 ± 0.08	
Plant associate traits	Rhizosphere	5 year	0.75 ± 0.31	0.255
		15 year	0.74 ± 0.33	
		35 year	0.18 ± 0.00	
		65 year	0.69 ± 0.32	
		105 year	0.85 ± 0.29	
	Endosphere	5 year	0.73 ± 0.39	0.189
		15 year	0.78 ± 0.33	
		35 year	0.77 ± 0.12	
		65 year	0.86 ± 0.25	
		105 year	0.75 ± 0.22	
Abiotic stress resistance	Rhizosphere	5 year	0.43 ± 0.16	0.459
		15 year	0.56 ± 0.25	
		35 year	0.50 ± 0.00	
		65 year	0.48 ± 0.15	
		105 year	0.40 ± 0.15	
	Endosphere	5 year	0.35 ± 0.00	0.364
		15 year	0.65 ± 0.24	
		35 year	0.74 ± 0.00	
		65 year	0.50 ± 0.24	
		105 year	0.51 ± 0.14	
Antibiotic resistance	Rhizosphere	5 year	0.47 ± 0.19	0.014^∗^
		15 year	0.32 ± 0.15	
		35 year	0.98 ± 0.00	
		65 year	0.43 ± 0.16	
		105 year	0.34 ± 0.15	
	Endosphere	5 year	0.26 ± 0.00	0.060
		15 year	0.68 ± 0.35	
		35 year	N.A.	
		65 year	0.66 ± 0.29	
		105 year	0.20 ± 0.08	
Metabolic potential	Rhizosphere	5 year	0.66 ± 0.22	0.995
		15 year	0.61 ± 0.38	
		35 year	0.71 ± 0.00	
		65 year	0.70 ± 0.28	
		105 year	0.66 ± 0.30	
	Endosphere	5 year	0.66 ± 0.32	0.071
		15 year	0.73 ± 0.26	
		35 year	N.A.	
		65 year	0.47 ± 0.29	
		105 year	0.69 ± 0.24	


*N.A., not applicable; ^∗^*P* < 0.05.*

**Table 2:** Comparison of functional diversity indices between two plant species.

Traits	Plant	Functional	*P*-value
	species	diversity	(K–W test)
Total	*Artemisia maritima*	0.76 ± 0.26	0.001^∗∗∗^
	*Limonium vulgare*	0.59 ± 0.27	
Plant associate traits	*Artemisia maritima*	0.83 ± 0.79	0.049^∗^
	*Limonium vulgare*	0.25 ± 0.31	
Abiotic stress resistance	*Artemisia maritima*	0.59 ± 0.20	0.004^∗∗^
	*Limonium vulgare*	0.55 ± 0.19	
Antibiotic resistance	*Artemisia maritima*	0.60 ± 0.26	0.388
	*Limonium vulgare*	0.58 ± 0.29	
Metabolic potential	*Artemisia maritima*	0.69 ± 0.30	0.001^∗∗∗^
	*Limonium vulgare*	0.30 ± 0.27	


*^∗^*P* < 0.05; ^∗∗^*P* < 0.01; ^∗∗∗^*P* < 0.001.*

## Discussion

The plant-associated microbiome is one of the key determinants of plant health and productivity (Daane et al., 2001; Penrose and Glick, 2003; Lugtenberg and Kamilova, 2009; Yang et al., 2009; Gayathri et al., 2010; Berg et al., 2014), playing an important role in plant phenotypic and epigenetic plasticity and further evolution (Partida-Martínez and Heil, 2011). It is therefore crucial to unravel the factors influencing the functionality of plant microbiome (Berg and Smalla, 2009; Berg et al., 2014), which varies in response to both soil properties and plant species (Garbeva et al., 2004b; Jousset et al., 2006; Berg and Smalla, 2009). Each plant species is thought to select specific microbial populations from soil through the driving force exerted by root exudates (Smalla et al., 2006; Faure et al., 2009; Cibichakravarthy et al., 2012). However, the plant selective force varies according to differences in agricultural practices, sampling sites or soil type, in order of importance (Garbeva et al., 2004b; Costa et al., 2006; Salles et al., 2006; Inceoğlu et al., 2010), although the effect of the latter is usually confounded with microbial biogeographical patterns. In this study, we disentangled the effect that soil and plant species exert on the functionality of bacteria isolated from rhizosphere and endosphere of two salt marsh plants, *L. vulgare* and *A. maritima*, by sampling along a natural gradient of soil formation that is exposed to the same microbial meta-community (Olff et al., 1997; Dini-Andreote et al., 2014, 2015; Wang et al., 2016). The absence of functional diversity pattern of isolates obtained from endosphere along the chronosequence would indicate plant selectivity to be stronger than the soil influence, whereas an increased complexity of functional diversity patterns associated with rhizosphere isolates along the succession would reveal an overriding effect of soil type over plant species.

### Taxonomic Distribution of Root-Associated Bacterial Isolates

From the taxonomic characteristics, most of the bacterial isolates were unique to one plant species or plant compartment (Supplementary Figure S2), which coincided with the effect of plant selectivity, recruiting unique microbes in response to the specific root exudates produced by different plants (Hartmann et al., 2009; Berendsen et al., 2012) and the degree of association with plant host, given the different biochemical environment observed in the internal root tissues (Gaiero et al., 2013; Timm et al., 2015). Specifically, this selectivity was obvious for the non-dominant groups — Bacilli, Bacteroidetes, Alpha- and Betaproteobacteria, probably because of the low number of isolated species associated with these classes. We observed a predominance of Gammaproteobacteria, especially belonging to the genus *Pseudomonas*, in our isolates despite the utilization of general isolation media. Similar results have been found elsewhere (Marilley and Aragno, 1999; Haas and Défago, 2005; Bharathkumar et al., 2008; Hong et al., 2013), which could be explained by the important functions that the genus *Pseudomonas* provides for plant hosts, such as plant growth promotion (Patten and Glick, 2002; Berg and Smalla, 2009; Rashid et al., 2012) and biological control (Chin-A-Woeng et al., 2003; Garbeva et al., 2004b; Beneduzi et al., 2012). The absolute predominance of Gammaproteobacteria from 65-year stage (Supplementary Figure S5) was also confirmed in our previous study on the rhizosphere of *L. vulgare* where more unique OTUs belonging to Gammaproteobacteria (a total of 99) were found at 65-year stage compared to other stages (Wang et al., 2016). These results could serve as an inference of a more stabilized community structure toward the end of succession, being in accordance with the reduced community turnover in rhizosphere (Wang et al., 2016), possibly resulting from the dominance of buffering effects of soil as well as plants following the development of succession (Walker and del Moral, 2003; Dini-Andreote et al., 2014). Despite the low number of individuals belonging to other classes, it is interesting to note that the highest diversity of other taxonomic groups was observed at the initial stage, which is subjected to a constant influx of different microbes owing to marine input (Dini-Andreote et al., 2014), followed by colonization in the different plant compartments.

### Functional Dynamics of the Rhizosphere Bacterial Isolates along the Chronosequence

The isolates obtained from the rhizosphere showed significant patterns along the chronosequence, specially for functions associated with bacterial fitness, indicating their response to soil properties as well as their potential to improve plant health. Specifically, the progressively increasing pattern of salinity stress resistance, antibiotic resistance and siderophore production of bacterial isolates were in line with the soil salinity accumulation and soil nutrients enrichment following the development of succession (Dini-Andreote et al., 2015; Wang et al., 2016). Moreover, it confirms the metagenomics data generated from bulk soil samples collected from the same field, where we observed an increase in genes associated with both salinity stress and antibiotic resistance at the end of the chronosequence (Dini-Andreote et al., 2017, submitted). These traits associated with antibiotic resistance are believed to play an important role in the adaptation of microbes to complex-structured environments like the soils found at the late successional stages. Despite the concurrence between studies, it is important to notice that the patterns observed in this study are obtained for a limited number of isolates and that an increase in the survey could lead to changes in the patterns. For instance, we expect that the difference between different plant compartments and plant species would be decreased, considering that more bacteria performing similar functions would be isolated. Below we discuss the most significant results obtained by grouping the bacterial isolates according to their specific functional diversity.

#### Bacterial Fitness (Antibiotic and Abiotic Stress Resistance)

In the context of salinity stress, the strains showing high resistance were isolated from the middle and late succession, and among them, *Pseudomonas* genus accounted for a large portion, which was consistent with the findings of Egamberdieva et al. (2008), who showed that the genus *Pseudomonas* and *Bacillus* could promote wheat growth in saline soils of Uzbekistan. Nadeem et al. (2016) also found that the rhizobacteria strain *Pseudomonas fluorescens* was the best strain for maintaining PGP traits under stressed conditions and alleviating the negative impacts of salinity on cucumber growth. Additionally, three *Serratia* sp. strains were also observed to show high salinity tolerance. This genus has been previously reported as capable of dealing with salt stress, which is the case of the strain *Serratia sp.* GSD2, which could sustain growth medium containing up to 10% NaCl, together with the increasing biofilm and extracellular enzyme production following the salinity increase (Mendpara et al., 2013). Regarding the resistance to biotic stress, strains belonging to genus *Serratia* showed high antibiotic resistance at late succession, which could indicate the general antifungal and antiparasitic properties often observed by this genus (Kalbe et al., 1996; Grimont and Grimont, 2006). Similarly, one *Pseudomonas* sp. strain from late stage also showed high antibiotic resistance, which was consistent with the high antagonistic activity found in other studies (Berg et al., 2002, 2006; Haas and Keel, 2003; Raaijmakers et al., 2010).

#### Plant Growth Promoting Capacity (Siderophore and Exoprotease Production, Biofilm Formation)

Siderophores are important traits associated with the plant microbiome, by competitively inhibiting the growth of plant pathogens or other harmful rhizosphere microbes with efficient Fe uptake system (Klopper, 1980; Sidhu et al., 1997; Bevivino et al., 1998; Maksimov et al., 2011; Gaiero et al., 2013; Ahmed and Holmström, 2014). Therefore, the increasing siderophore production observed in the rhizosphere isolates along the chronosequence may be also as a result of the shifts in microbial composition following the development of succession (Dini-Andreote et al., 2014, 2016), which might be associated with higher microbial competition, as siderophore production could confer competitive advantages to rhizosphere-associated bacteria (Haas and Défago, 2005). Three *Pseudomonas* sp. strains were found to produce relatively more siderophores among others, which was confirmed by many studies related to siderophore production by *Pseudomonas* genus for plant growth promotion or biocontrol function against plant pathogen (Crosa, 1997; Dwivedi and Johri, 2003; Sharma and Johri, 2003; Sasirekha and Shivakumar, 2016). A pseudobactin siderophore produced by *P. putida* B10 strain was able to suppress *Fusarium oxysporum* in iron-deficient soil (Klopper, 1980). Additionally, Bar-Ness et al. (1991) found that ferrated (^59^Fe) pseudobactin (PSB) (^59^FePSB) — a *Pseudomonas putida* siderophore could be more efficiently assimilated by plants compared with FeEDDHA, which was also proved by other studies (Masalha et al., 2000; Katiyar and Goel, 2004; Dimkpa et al., 2009).

### Functional Dynamics of the Endosphere Bacterial Isolates along the Chronosequence

Contrary to the rhizosphere isolates, the majority of functions associated with endophytes, except for resistance to salinity and osmotic stress, remained constant along the chronosequence, confirming our hypothesis that plant selectivity would be stronger in these communities. Thus, the selectivity and the buffering effect offered by plants against abiotic and biotic stresses (Hallmann et al., 1997; Rosenblueth and Martínez-Romero, 2006; Schulz and Boyle, 2006) lead to a stable community structure (Wang et al., 2016) and further functional profile for the root endophytes. Interestingly, the resistance against salinity and osmotic stress dramatically increased at the 105-year stage, which was in line with the community turnover of endophytes observed earlier when assessing *L. vulgare*-associated bacterial communities through molecular methods (Wang et al., 2016). This could be derived from the stress experienced by both *L. vulgare* and *A. maritima* because of the higher salinity level and severe competition with other plants at the late stage, specially *Elytrigia atherica*, which dominates the vegetation (Schrama et al., 2012). The reason why these two traits were synchronized in response to the soil and plant changes along the succession could be explained by the inhibitory effect of salt on plant growth, causing both ion toxicity and osmotic stress (Zhang et al., 2010; Tavakkoli et al., 2011).

### Functional Diversity of the Plant Microbiome

By disentangling the distributional pattern of functional traits from different treatments and by grouping the functional traits into an overall functional diversity measure we observed that the functional diversity of bacterial isolates was highest at intermediate stages of succession. We could speculate that the intermediate soil nutritional level, intermediate stressful abiotic (salinity, higher flooding frequency) and biotic environment (plant competition) at the stage could lead to a selection of more versatile root-associated bacteria that can better adapt to and cope with the variety of biotic and abiotic conditions, eventually leading to an enrichment of functional diversity in the intermediate succession. This high functional diversity was, however, not associated with the taxonomic diversity, which was highest at early stages of succession. The screening of a larger number of isolates should provide a better characterization of the potential (lack of) link between taxonomy and function, which should be the subject of follow up studies. For instance, the screening of larger number of strains from the initial stage could eventually increase the functional diversity patterns, which should lead to a larger dispersion in **Figure 3**. However, given the low functional variation observed despite the high taxonomic diversity, we argue that is not likely to occur. Likewise, the screening a larger number of isolates should not modify the observed high functional diversity assigned to intermediate stage. The lowest functional diversity was observed in the 105-year stage, characterized by high soil nutrient content, plant biomass and low environmental fluctuations. We speculate that this low variability in abiotic and biotic stress lead to a more stable, functionally specialized microbiome. We expect that this pattern would remain the same if more isolates would be screened, given that the bacterial isolates in the dominant taxonomic group at this stage are closely related in phylogeny. Additionally, the plant species exerted an influence on the root-associated bacterial functional diversity which was higher in the *A. maritima* microbiome, which mainly derived from the difference of root exudates from different plants (Hartmann et al., 2009; Bednarek et al., 2010; Berendsen et al., 2012), resulting in plant specificity of microbial communities (Haichar et al., 2008; Berg and Smalla, 2009; Turner et al., 2013; Ofek-Lalzar et al., 2014).

## Conclusion

Overall, this study provides key information on the variation in functionality of bacterial isolates associated with rhizosphere and endosphere of *L. vulgare* and *A. maritima* along a salt marsh primary succession chronosequence. We could show that the importance of soil in driving the functional diversity of root-associated bacterial isolates is dependent on the plant compartments, being influential only in the rhizosphere but not on the endosphere. Interestingly, the intermediate successional stage exhibits the highest functional trait diversity associated with antibiotics resistance of rhizobacterial isolates. While the constant distribution of functional diversity of endosphere isolates indicates a strong plant selective force and buffering effect on endophytes. Specifically, we could show that some *Pseudomonas* sp. and *Serratia* sp. strains reveal high resistance to abiotic stress and antibiotics and produce more siderophores, confirming the high PGP activity of these two genera. Additionally, the distinctive distribution of taxonomic groups according to specific plant compartment or plant species validates the existence of the plant selective force on the root-associated microbes, resulting in the distinctive taxonomy composition and diversity in the rhizosphere and the internal root tissues and among different plant species.

## Author Contributions

MW participated in study design, took samples, did the experiments and write the manuscript. EL contributed to the modified methods for biochemical tests in this study. CL helped with the main experimental part of biochemical tests. AJ provided his lab for conducting the biochemical tests and discussed about the results in the manuscript. JS supervised the project and participated in the decision of every part in the manuscript.

## Conflict of Interest Statement

The authors declare that the research was conducted in the absence of any commercial or financial relationships that could be construed as a potential conflict of interest.

## Footnotes

1 https://blast.ncbi.nlm.nih.gov/Blast.cgi

2 http://www.r-project.org
