# Supplementary material for: Functionality of Root-Associated Bacteria along a Salt Marsh Primary Succession
Source: Front Microbiol. 2017 Oct 30;8:2102. doi: 10.3389/fmicb.2017.02102 (PMC5670159; doi:10.3389/fmicb.2017.02102)
Supplement: Supplementary file 1 [file Table_1.DOCX]

**Table S1 Screening procedure for the bacterial isolates from R2A agar plate**

| **Plate ID** | **Source** | **Plant species** | **Stage** | **Dilution (log_10_)** | **Total number of colonies on each plate** | **Total number of selected colonies with unique morphologies from each plate** | **Number of Box patterns** | **BOX pattern ID** | **Number of colonies** |
| --- | --- | --- | --- | --- | --- | --- | --- | --- | --- |
| 1 | Rhizosphere | *Limonium vulgare* | 5y | -3 | 165 | 32 | 5 | 1 | 8 |
|  |  |  |  |  |  |  |  | 2 | 6 |
|  |  |  |  |  |  |  |  | 3 | 6 |
|  |  |  |  |  |  |  |  | 4 | 7 |
|  |  |  |  |  |  |  |  | 5 | 5 |
| 2 | Rhizosphere | *Limonium vulgare* | 5y | -3 | 396 | 31 | 4 | 6 | 4 |
|  |  |  |  |  |  |  |  | 7 | 10 |
|  |  |  |  |  |  |  |  | 8 | 12 |
|  |  |  |  |  |  |  |  | 9 | 5 |
| 3 | Rhizosphere | *Limonium vulgare* | 5y | -3 | 239 | 31 | 2 | 10 | 9 |
|  |  |  |  |  |  |  |  | 11 | 22 |
| 4 | Rhizosphere | *Limonium vulgare* | 15y | -2 | 26 | 20 | 3 | 12 | 8 |
|  |  |  |  |  |  |  |  | 13 | 6 |
|  |  |  |  |  |  |  |  | 14 | 6 |
| 5 | Rhizosphere | *Limonium vulgare* | 15y | -3 | 188 | 16 | 2 | 15 | 10 |
|  |  |  |  |  |  |  |  | 16 | 6 |
| 6 | Rhizosphere | *Limonium vulgare* | 65y | -2 | 74 | 34 | 6 | 17 | 3 |
|  |  |  |  |  |  |  |  | 18 | 5 |
|  |  |  |  |  |  |  |  | 19 | 4 |
|  |  |  |  |  |  |  |  | 20 | 5 |
|  |  |  |  |  |  |  |  | 21 | 8 |
|  |  |  |  |  |  |  |  | 22 | 9 |
| 7 | Rhizosphere | *Limonium vulgare* | 105y | -3 | 211 | 20 | 6 | 23 | 6 |
|  |  |  |  |  |  |  |  | 24 | 2 |
|  |  |  |  |  |  |  |  | 25 | 3 |
|  |  |  |  |  |  |  |  | 26 | 2 |
|  |  |  |  |  |  |  |  | 27 | 2 |
|  |  |  |  |  |  |  |  | 28 | 5 |
| 8 | Rhizosphere | *Limonium vulgare* | 105y | -3 | 418 | 31 | 3 | 29 | 8 |
|  |  |  |  |  |  |  |  | 30 | 7 |
|  |  |  |  |  |  |  |  | 31 | 16 |
| 9 | Rhizosphere | *Limonium vulgare* | 105y | -3 | 152 | 23 | 4 | 32 | 6 |
|  |  |  |  |  |  |  |  | 33 | 6 |
|  |  |  |  |  |  |  |  | 34 | 9 |
|  |  |  |  |  |  |  |  | 35 | 2 |
| 10 | Rhizosphere | *Artemisia maritima* | 5y | -3 | 129 | 15 | 3 | 36 | 3 |
|  |  |  |  |  |  |  |  | 37 | 6 |
|  |  |  |  |  |  |  |  | 38 | 6 |
| 11 | Rhizosphere | *Artemisia maritima* | 5y | -3 | 157 | 22 | 2 | 39 | 10 |
|  |  |  |  |  |  |  |  | 40 | 12 |
| 12 | Rhizosphere | *Artemisia maritima* | 5y | -3 | 116 | 22 | 2 | 41 | 12 |
|  |  |  |  |  |  |  |  | 42 | 10 |
| 13 | Rhizosphere | *Artemisia maritima* | 15y | -3 | 182 | 16 | 4 | 43 | 3 |
|  |  |  |  |  |  |  |  | 44 | 4 |
|  |  |  |  |  |  |  |  | 45 | 5 |
|  |  |  |  |  |  |  |  | 46 | 4 |
| 14 | Rhizosphere | *Artemisia maritima* | 15y | -2 | 209 | 33 | 9 | 47 | 2 |
|  |  |  |  |  |  |  |  | 48 | 3 |
|  |  |  |  |  |  |  |  | 49 | 4 |
|  |  |  |  |  |  |  |  | 50 | 3 |
|  |  |  |  |  |  |  |  | 51 | 7 |
|  |  |  |  |  |  |  |  | 52 | 4 |
|  |  |  |  |  |  |  |  | 53 | 3 |
|  |  |  |  |  |  |  |  | 54 | 3 |
|  |  |  |  |  |  |  |  | 55 | 4 |
| 15 | Rhizosphere | *Artemisia maritima* | 15y | -2 | 188 | 19 | 5 | 56 | 3 |
|  |  |  |  |  |  |  |  | 57 | 3 |
|  |  |  |  |  |  |  |  | 58 | 3 |
|  |  |  |  |  |  |  |  | 59 | 5 |
|  |  |  |  |  |  |  |  | 60 | 5 |
| 16 | Rhizosphere | *Artemisia maritima* | 35y | -2 | 156 | 24 | 3 | 61 | 8 |
|  |  |  |  |  |  |  |  | 62 | 10 |
|  |  |  |  |  |  |  |  | 63 | 6 |
| 17 | Rhizosphere | *Artemisia maritima* | 35y | -1 | 60 | 31 | 5 | 64 | 3 |
|  |  |  |  |  |  |  |  | 65 | 6 |
|  |  |  |  |  |  |  |  | 66 | 5 |
|  |  |  |  |  |  |  |  | 67 | 10 |
|  |  |  |  |  |  |  |  | 68 | 7 |
| 18 | Rhizosphere | *Artemisia maritima* | 65y | -1 | 60 | 31 | 2 | 69 | 16 |
|  |  |  |  |  |  |  |  | 70 | 15 |
| 19 | Rhizosphere | *Artemisia maritima* | 105y | -3 | 143 | 20 | 4 | 71 | 8 |
|  |  |  |  |  |  |  |  | 72 | 4 |
|  |  |  |  |  |  |  |  | 73 | 3 |
|  |  |  |  |  |  |  |  | 74 | 5 |
| 20 | Rhizosphere | *Artemisia maritima* | 105y | -3 | 600 | 29 | 3 | 75 | 6 |
|  |  |  |  |  |  |  |  | 76 | 7 |
|  |  |  |  |  |  |  |  | 77 | 16 |
| 21 | Endosphere | *Limonium vulgare* | 5y | -3 | 72 | 11 | 3 | 78 | 3 |
|  |  |  |  |  |  |  |  | 79 | 3 |
|  |  |  |  |  |  |  |  | 80 | 2 |
| 22 | Endosphere | *Limonium vulgare* | 5y | -3 | 212 | 13 | 3 | 81 | 4 |
|  |  |  |  |  |  |  |  | 82 | 6 |
|  |  |  |  |  |  |  |  | 83 | 3 |
| 23 | Endosphere | *Limonium vulgare* | 5y | -3 | 206 | 21 | 6 | 84 | 3 |
|  |  |  |  |  |  |  |  | 85 | 3 |
|  |  |  |  |  |  |  |  | 86 | 2 |
|  |  |  |  |  |  |  |  | 87 | 2 |
|  |  |  |  |  |  |  |  | 88 | 6 |
|  |  |  |  |  |  |  |  | 89 | 5 |
| 24 | Endosphere | *Limonium vulgare* | 15y | -2 | 119 | 12 | 3 | 90 | 5 |
|  |  |  |  |  |  |  |  | 91 | 4 |
|  |  |  |  |  |  |  |  | 92 | 3 |
| 25 | Endosphere | *Limonium vulgare* | 15y | -2 | 291 | 9 | 2 | 93 | 4 |
|  |  |  |  |  |  |  |  | 94 | 5 |
| 26 | Endosphere | *Limonium vulgare* | 35y | -2 | 170 | 8 | 3 | 95 | 3 |
|  |  |  |  |  |  |  |  | 96 | 3 |
|  |  |  |  |  |  |  |  | 97 | 2 |
| 27 | Endosphere | *Limonium vulgare* | 35y | -3 | 89 | 2 | 1 | 98 | 2 |
| 28 | Endosphere | *Limonium vulgare* | 65y | -1 | 84 | 18 | 4 | 99 | 6 |
|  |  |  |  |  |  |  |  | 100 | 4 |
|  |  |  |  |  |  |  |  | 101 | 4 |
|  |  |  |  |  |  |  |  | 102 | 4 |
| 29 | Endosphere | *Limonium vulgare* | 65y | -2 | 242 | 15 | 4 | 103 | 3 |
|  |  |  |  |  |  |  |  | 104 | 5 |
|  |  |  |  |  |  |  |  | 105 | 3 |
|  |  |  |  |  |  |  |  | 106 | 4 |
| 30 | Endosphere | *Limonium vulgare* | 65y | -2 | 110 | 10 | 3 | 107 | 3 |
|  |  |  |  |  |  |  |  | 108 | 3 |
|  |  |  |  |  |  |  |  | 109 | 4 |
| 31 | Endosphere | *Limonium vulgare* | 105y | -1 | 19 | 12 | 3 | 110 | 4 |
|  |  |  |  |  |  |  |  | 111 | 4 |
|  |  |  |  |  |  |  |  | 112 | 4 |
| 32 | Endosphere | *Limonium vulgare* | 105y | -3 | 360 | 12 | 3 | 113 | 3 |
|  |  |  |  |  |  |  |  | 114 | 4 |
|  |  |  |  |  |  |  |  | 115 | 5 |
| 33 | Endosphere | *Artemisia maritima* | 5y | -3 | 295 | 8 | 3 | 116 | 2 |
|  |  |  |  |  |  |  |  | 117 | 3 |
|  |  |  |  |  |  |  |  | 118 | 3 |
| 34 | Endosphere | *Artemisia maritima* | 5y | -3 | 47 | 14 | 3 | 119 | 5 |
|  |  |  |  |  |  |  |  | 120 | 6 |
|  |  |  |  |  |  |  |  | 121 | 3 |
| 35 | Endosphere | *Artemisia maritima* | 15y | -3 | 294 | 7 | 2 | 122 | 4 |
|  |  |  |  |  |  |  |  | 123 | 3 |
| 36 | Endosphere | *Artemisia maritima* | 15y | -2 | 208 | 6 | 2 | 124 | 3 |
|  |  |  |  |  |  |  |  | 125 | 3 |
| 37 | Endosphere | *Artemisia maritima* | 15y | -2 | 170 | 12 | 3 | 126 | 4 |
|  |  |  |  |  |  |  |  | 127 | 4 |
|  |  |  |  |  |  |  |  | 128 | 4 |
| 38 | Endosphere | *Artemisia maritima* | 35y | -3 | 89 | 15 | 4 | 129 | 3 |
|  |  |  |  |  |  |  |  | 130 | 3 |
|  |  |  |  |  |  |  |  | 131 | 6 |
|  |  |  |  |  |  |  |  | 132 | 3 |
| 39 | Endosphere | *Artemisia maritima* | 35y | -2 | 52 | 15 | 4 | 133 | 4 |
|  |  |  |  |  |  |  |  | 134 | 4 |
|  |  |  |  |  |  |  |  | 135 | 4 |
|  |  |  |  |  |  |  |  | 136 | 3 |
| 40 | Endosphere | *Artemisia maritima* | 65y | -3 | 47 | 14 | 4 | 137 | 3 |
|  |  |  |  |  |  |  |  | 138 | 3 |
|  |  |  |  |  |  |  |  | 139 | 4 |
|  |  |  |  |  |  |  |  | 140 | 4 |
| 41 | Endosphere | *Artemisia maritima* | 65y | -3 | 73 | 12 | 4 | 141 | 4 |
|  |  |  |  |  |  |  |  | 142 | 2 |
|  |  |  |  |  |  |  |  | 143 | 3 |
|  |  |  |  |  |  |  |  | 144 | 3 |
| 42 | Endosphere | *Artemisia maritima* | 65y | -1 | 400 | 18 | 5 | 145 | 4 |
|  |  |  |  |  |  |  |  | 146 | 4 |
|  |  |  |  |  |  |  |  | 147 | 3 |
|  |  |  |  |  |  |  |  | 148 | 3 |
|  |  |  |  |  |  |  |  | 149 | 4 |
| 43 | Endosphere | *Artemisia maritima* | 105y | -2 | 77 | 21 | 4 | 150 | 6 |
|  |  |  |  |  |  |  |  | 151 | 5 |
|  |  |  |  |  |  |  |  | 152 | 5 |
|  |  |  |  |  |  |  |  | 153 | 5 |
| 44 | Endosphere | *Artemisia maritima* | 105y | -3 | 600 | 11 | 3 | 154 | 5 |
|  |  |  |  |  |  |  |  | 155 | 3 |
|  |  |  |  |  |  |  |  | 156 | 3 |
| 45 | Endosphere | *Artemisia maritima* | 105y | -3 | 990 | 12 | 3 | 157 | 4 |
|  |  |  |  |  |  |  |  | 158 | 4 |
|  |  |  |  |  |  |  |  | 159 | 4 |
