# Supplementary material for: Functionality of Root-Associated Bacteria along a Salt Marsh Primary Succession
Source: Front Microbiol. 2017 Oct 30;8:2102. doi: 10.3389/fmicb.2017.02102 (PMC5670159; doi:10.3389/fmicb.2017.02102)
Supplement: Supplementary file 2 [file Table_2.DOCX]

**Table S2 Summary of bacterial isolates from different plants, sources and successional stages**

| **Plant species** | **Sources** | **Stage** | **Total number of screnned colonies 1** | **Total number of strains 2** | **Bacterial species 3** | **Number of BOX-PCR patterns 4** | **weight 5** |
| --- | --- | --- | --- | --- | --- | --- | --- |
| *Limonium vulgare* | Rhizosphere | 5y | 94 | 8 | *Arthrobacter sp. Aza6* | 1 | 0.09 |
|  |  |  |  |  | *Arthrobacter aurescens strain GEM(1)* | 1 | 0.06 |
|  |  |  |  |  | *Pseudomonas fluorescens strain FW300-N2E2* | 2 | 0.14 |
|  |  |  |  |  | *Pseudomonas psychrophila strain PP02* | 2 | 0.1 |
|  |  |  |  |  | *Pseudomonas chlororaphis strain UFB2* | 1 | 0.11 |
|  |  |  |  |  | *Kluyvera intermedia strain MLS-6-8* | 1 | 0.13 |
|  |  |  |  |  | *Erwinia sp. CanR-65* | 1 | 0.05 |
|  |  |  |  |  | *Pseudomonas sp. UT 6-06* | 2 | 0.33 |
| *Limonium vulgare* | Rhizosphere | 15y | 36 | 1 | *Enterobacteriaceae bacterium SR5* | 6 | 1 |
| *Limonium vulgare* | Rhizosphere | 65y | 34 | 3 | *Serratia plymuthica strain I-A-E-24* | 1 | 0.09 |
|  |  |  |  |  | *Pseudomonas sp. CanL-3* | 3 | 0.41 |
|  |  |  |  |  | *Pseudomonas putida AV4* | 2 | 0.5 |
| *Limonium vulgare* | Rhizosphere | 105y | 74 | 5 | *Hafnia psychrotolerans strain CSE_16* | 3 | 0.15 |
|  |  |  |  |  | *Pseudomonas sp. 332* | 4 | 0.23 |
|  |  |  |  |  | *Pseudomonas sp. SB11* | 2 | 0.31 |
|  |  |  |  |  | *Erwinia rhapontici strain A534* | 2 | 0.16 |
|  |  |  |  |  | *Pseudomonas sp. strain JWp16* | 2 | 0.15 |
| *Artemisia maritima* | Rhizosphere | 5y | 59 | 7 | *Arthrobacter nitroguajacolicus strain GEM(1)* | 1 | 0.05 |
|  |  |  |  |  | *Pseudomonas sp. 19K3G2* | 1 | 0.1 |
|  |  |  |  |  | *Sphingobacterium faecium strain NBRC 15337* | 1 | 0.1 |
|  |  |  |  |  | *Flavobacterium frigidimaris strain BK22* | 1 | 0.17 |
|  |  |  |  |  | *Pseudomonas sp. JY-Q* | 1 | 0.2 |
|  |  |  |  |  | *Flavobacterium sp. FLX-4* | 1 | 0.2 |
|  |  |  |  |  | *Flavobacterium sp. WB4.4-97* | 1 | 0.17 |
| *Artemisia maritima* | Rhizosphere | 15y | 68 | 7 | *Pseudomonas sp. ARCTIC-P37* | 9 | 0.51 |
|  |  |  |  |  | *Psychrobacter alimentarius strain B1.2* | 1 | 0.06 |
|  |  |  |  |  | *Psychrobacter sp. A-1-45* | 2 | 0.09 |
|  |  |  |  |  | *Serratia fonticola strain 51* | 3 | 0.15 |
|  |  |  |  |  | *Bacillus simplex strain NA-4* | 1 | 0.04 |
|  |  |  |  |  | *Pantoea sp. LB-397-GYM-5* | 1 | 0.07 |
|  |  |  |  |  | *Serratia rubidaea strain D52* | 1 | 0.07 |
| *Artemisia maritima* | Rhizosphere | 35y | 55 | 2 | *Microbacterium sp. S1(2016)* | 4 | 0.49 |
|  |  |  |  |  | *Psychrobacter sp. EB231* | 4 | 0.51 |
| *Artemisia maritima* | Rhizosphere | 65y | 31 | 2 | *Serratia plymuthica strain NBRC 102599* | 1 | 0.52 |
|  |  |  |  |  | *Stenotrophomonas maltophilia strain ATCC 13676* | 1 | 0.48 |
| *Artemisia maritima* | Rhizosphere | 105y | 49 | 4 | *Exiguobacterium oxidotolerans strain LPB0102* | 1 | 0.16 |
|  |  |  |  |  | *Microbacterium oxydans strain PMR64* | 1 | 0.08 |
|  |  |  |  |  | *Rahnella aquatilis strain NA06* | 1 | 0.06 |
|  |  |  |  |  | *Pseudomonas sp. 46(2016)* | 4 | 0.69 |
| *Limonium vulgare* | Endosphere | 5y | 42 | 6 | *Pseudomonas sp. L10.10* | 2 | 0.14 |
|  |  |  |  |  | *Hafnia psychrotolerans strain CSE_16* | 2 | 0.14 |
|  |  |  |  |  | *Albirhodobacter sp. S1-47* | 1 | 0.14 |
|  |  |  |  |  | *Pseudomonas sp. 332* | 3 | 0.21 |
|  |  |  |  |  | *Pseudomonas sp. 291(2016)* | 3 | 0.24 |
|  |  |  |  |  | *Janthinobacterium lividum strain CH1-13* | 1 | 0.12 |
| *Limonium vulgare* | Endosphere | 15y | 21 | 4 | *Pseudomonas brassicacearum strain SAS16* | 1 | 0.24 |
|  |  |  |  |  | *Pseudomonas sp. 46(2016)* | 2 | 0.33 |
|  |  |  |  |  | *Rheinheimera sp. DS5* | 1 | 0.19 |
|  |  |  |  |  | *Pseudomonas sp. XH1* | 1 | 0.24 |
| *Limonium vulgare* | Endosphere | 35y | 10 | 4 | *Pseudomonas sp. R76* | 1 | 0.3 |
|  |  |  |  |  | *Rahnella sp. UIWRF1115* | 1 | 0.3 |
|  |  |  |  |  | *Serratia fonticola strain 51* | 1 | 0.2 |
|  |  |  |  |  | *Pseudomonas sp. T7C* | 1 | 0.2 |
| *Limonium vulgare* | Endosphere | 65y | 43 | 7 | *Pseudomonas sp. FE4* | 1 | 0.14 |
|  |  |  |  |  | *Pseudomonas sp. RZ110* | 2 | 0.19 |
|  |  |  |  |  | *Erwinia persicina B57* | 1 | 0.09 |
|  |  |  |  |  | *Pseudomonas sp. DSM 29166* | 2 | 0.19 |
|  |  |  |  |  | *Pseudomonas sp. CanL-3* | 2 | 0.16 |
|  |  |  |  |  | *Pseudomonas sp. GR 6-02* | 2 | 0.14 |
|  |  |  |  |  | *Pseudomonas sp. CH2(2014)* | 1 | 0.09 |
| *Limonium vulgare* | Endosphere | 105y | 24 | 1 | *Pseudomonas putida strain S28* | 6 | 1 |
| *Artemisia maritima* | Endosphere | 5y | 22 | 2 | *Pseudomonas sp. UT 6-06* | 4 | 0.59 |
|  |  |  |  |  | *Pseudomonas fluorescens strain hpa0041* | 2 | 0.41 |
| *Artemisia maritima* | Endosphere | 15y | 25 | 3 | *Pseudomonas sp. TFS130* | 1 | 0.16 |
|  |  |  |  |  | *Pseudomonas sp. JY-Q* | 3 | 0.36 |
|  |  |  |  |  | *Serratia sp. B37/06* | 3 | 0.48 |
| *Artemisia maritima* | Endosphere | 35y | 30 | 2 | *Pseudomonas sp. RZ16* | 3 | 0.4 |
|  |  |  |  |  | *Pseudomonas sp. 389* | 4 | 0.6 |
| *Artemisia maritima* | Endosphere | 65y | 44 | 7 | *Pseudomonas sp. 12Kp11* | 1 | 0.07 |
|  |  |  |  |  | *Pseudomonas sp. ARCTIC-P37* | 4 | 0.34 |
|  |  |  |  |  | *Pseudomonas fluorescens strain FW300-N2E2* | 3 | 0.18 |
|  |  |  |  |  | *Serratia plymuthica PRI-2C* | 2 | 0.18 |
|  |  |  |  |  | *Microbacterium foliorum YIM130897* | 1 | 0.07 |
|  |  |  |  |  | *Erwinia sp. strain SH18* | 1 | 0.07 |
|  |  |  |  |  | *Stenotrophomonas sp. PDD-61b-6* | 1 | 0.09 |
| *Artemisia maritima* | Endosphere | 105y | 44 | 2 | *Pseudomonas sp. BWDY-40* | 5 | 0.59 |
|  |  |  |  |  | *Pantoea sp. FB22012* | 5 | 0.41 |

**1. Total number of colonies with unique morphologies, isolated using R2A agar plate;**

**2. Colonies with identical BOX-PCR patterns were assigned as identical strains;**

**3. Bacterial identity was determined by sequencing the 16S rRNA gene;**

**4. Number of unique BOX-patterns that were assigned to each bacterial species;**

**5. Weighted was calculated by the ratio of the number of colonies identified as a specific strain to the total number of colonies obtained from the corresponding treatment. The weight was used to normalize the biochemical data, so that it represented the relative abundance of each species in the samples.**
