# Supplementary material for: Functionality of Root-Associated Bacteria along a Salt Marsh Primary Succession
Source: Front Microbiol. 2017 Oct 30;8:2102. doi: 10.3389/fmicb.2017.02102 (PMC5670159; doi:10.3389/fmicb.2017.02102)
Supplement: Supplementary file 3 [file Table_3.DOCX]

**Table S3 Accession numbers of bacterial strains**

| Bacterial strains | Accession number |
| --- | --- |
| *Pseudomonas sp. ARCTIC-P37* | MF664115 |
| *Arthrobacter sp. Aza6* | MF664116 |
| *Arthrobacter aurescens strain GEM(1)* | MF664117 |
| *Serratia plymuthica strain I-A-E-24* | MF664118 |
| *Arthrobacter nitroguajacolicus strain 33-04(1)* | MF664119 |
| *Pseudomonas sp. 19K3G2* | MF664120 |
| *Hafnia psychrotolerans strain CSE_16* | MF664121 |
| *Microbacterium sp. S1(2016)* | MF664122 |
| *Pseudomonas fluorescens strain FW300-N2E2* | MF664123 |
| *Pseudomonas psychrophila strain PP02* | MF664124 |
| *Pseudomonas sp. CanL-3* | MF664125 |
| *Sphingobacterium faecium* | MF664126 |
| *Flavobacterium frigidimaris strain BK22* | MF664127 |
| *Pseudomonas chlororaphis strain UFB2* | MF664128 |
| *Pseudomonas sp. 332* | MF664129 |
| *Exiguobacterium oxidotolerans strain LPB0102* | MF664130 |
| *Psychrobacter alimentarius strain B1.2* | MF664131 |
| *Pseudomonas sp. JY-Q* | MF664132 |
| *Psychrobacter sp. A-1-45* | MF664133 |
| *Psychrobacter sp. EB231* | MF664134 |
| *Flavobacterium sp. FLX-4* | MF664135 |
| *Pseudomonas sp. SB11* | MF664136 |
| *Microbacterium oxydans strain PMR64* | MF664137 |
| *Serratia fonticola strain 51* | MF664138 |
| *Rahnella aquatilis strain NA06* | MF664139 |
| *Enterobacteriaceae bacterium SR5* | MF664140 |
| *Bacillus simplex strain NA-4* | MF664141 |
| *Pantoea sp. LB-397-GYM-5* | MF664142 |
| *Pseudomonas putida strain AV4* | MF664143 |
| *Pseudomonas sp. 46(2016)* | MF664144 |
| *Kluyvera intermedia strain MLS-6-8* | MF664145 |
| *Erwinia sp. CanR-65* | MF664146 |
| *Serratia rubidaea strain D52* | MF664147 |
| *Serratia sp. B37/06* | MF664148 |
| *Pseudomonas sp. UT 6-06* | MF664149 |
| *Stenotrophomonas maltophilia strain ATCC 13637* | MF664150 |
| *Erwinia rhapontici strain A534* | MF664151 |
| *Pseudomonas sp. JWp16* | MF664152 |
| *Pseudomonas sp. BWDY-40* | MF664153 |
| *Pseudomonas brassicacearum strain SAS16* | MF664154 |
| *Pseudomonas sp. 12Kp11* | MF664155 |
| *Pseudomonas sp. RZ16* | MF664156 |
| *Pantoea sp. FB22012* | MF664157 |
| *Pseudomonas sp. L10.10* | MF664158 |
| *Albirhodobacter sp. S1-47* | MF664159 |
| *Pseudomonas sp. FE4* | MF664160 |
| *Pseudomonas sp. R76* | MF664161 |
| *Pseudomonas sp. RZ110* | MF664162 |
| *Rahnella sp. UIWRF1115* | MF664163 |
| *Pseudomonas sp. 291(2016)* | MF664164 |
| *Erwinia persicina* | MF664165 |
| *Pseudomonas sp. TFS130* | MF664166 |
| *Rheinheimera sp. DS5* | MF664167 |
| *Serratia plymuthica PRI-2C* | MF664168 |
| *Pseudomonas sp. XH1* | MF664169 |
| *Pseudomonas sp. T7C* | MF664170 |
| *Pseudomonas putida strain S28* | MF664171 |
| *Pseudomonas fluorescens strain hpa0041* | MF664172 |
| *Pseudomonas sp. GR 6-02* | MF664173 |
| *Janthinobacterium lividum strain CH1-13* | MF664174 |
| *Pseudomonas sp. CH2(2014)* | MF664175 |
| *Pseudomonas sp. 389* | MF664176 |
| *Microbacterium foliorum strain YIM 130897* | MF664177 |
| *Erwinia sp. SH18* | MF664178 |
| *Stenotrophomonas sp. PDD-61b-6* | MF664179 |
| *Serratia plymuthicaNBRC 102599* | MF677865 |
| *Pseudomonas sp. DSM29166* | MF677866 |
| *Flavobacterium sp. WB4.4-97* | MF677867 |
