# Supplementary material for: Functionality of Root-Associated Bacteria along a Salt Marsh Primary Succession
Source: Front Microbiol. 2017 Oct 30;8:2102. doi: 10.3389/fmicb.2017.02102 (PMC5670159; doi:10.3389/fmicb.2017.02102)
Supplement: Supplementary file 4 [file Presentation_1.PDF]

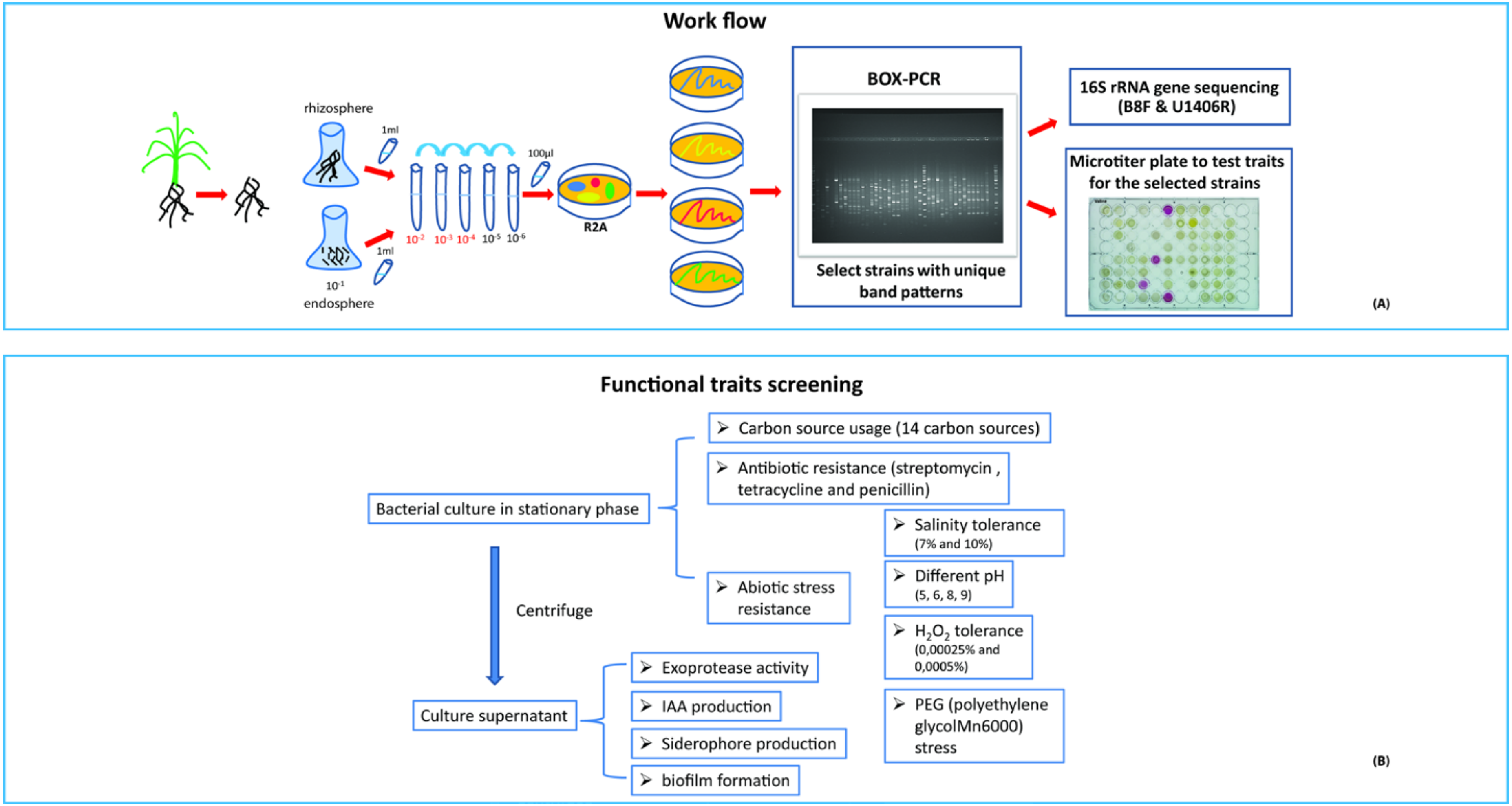

Figure S1 Isolating, screening and charaterizing root-associated bacterial isolates. (A) Work flow of isolating, molecular charaterization and biochemical screening of root-associated bacteria isolates, (B) Biochemical assays for functional traits Screening.

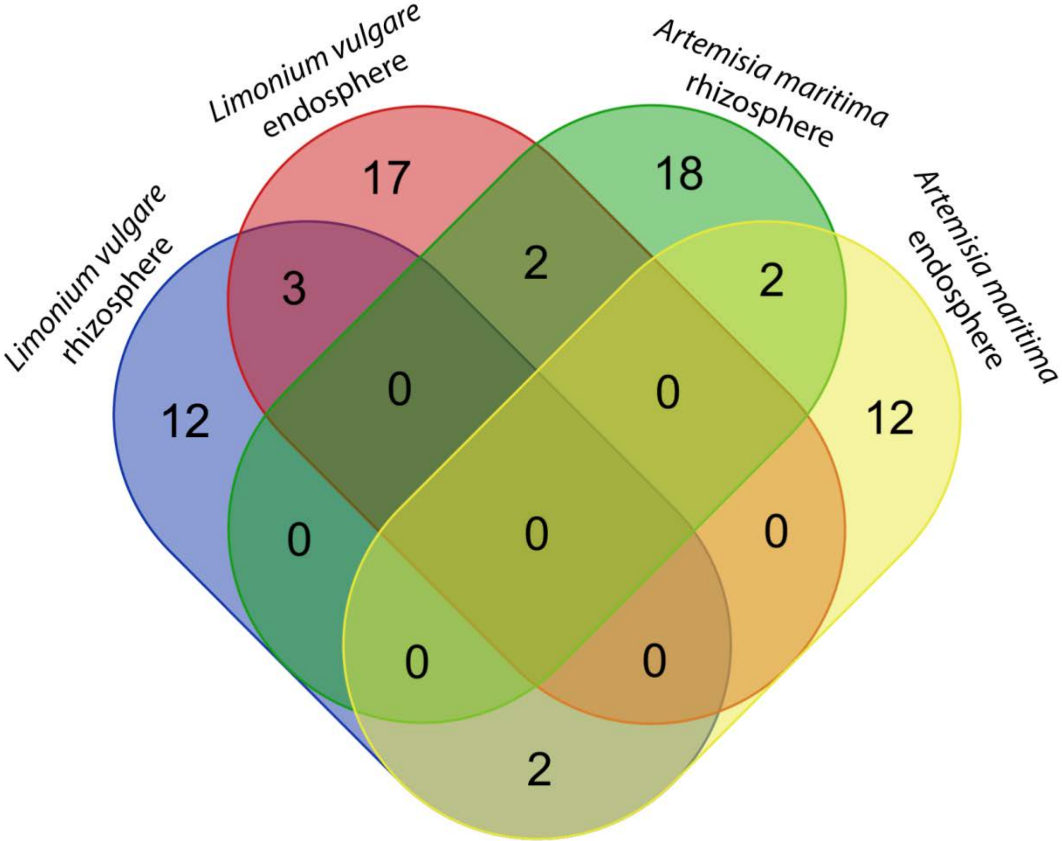

Figure S2 Venn diagram of bacterial isolates distribution in different plant compartments from different plant species.

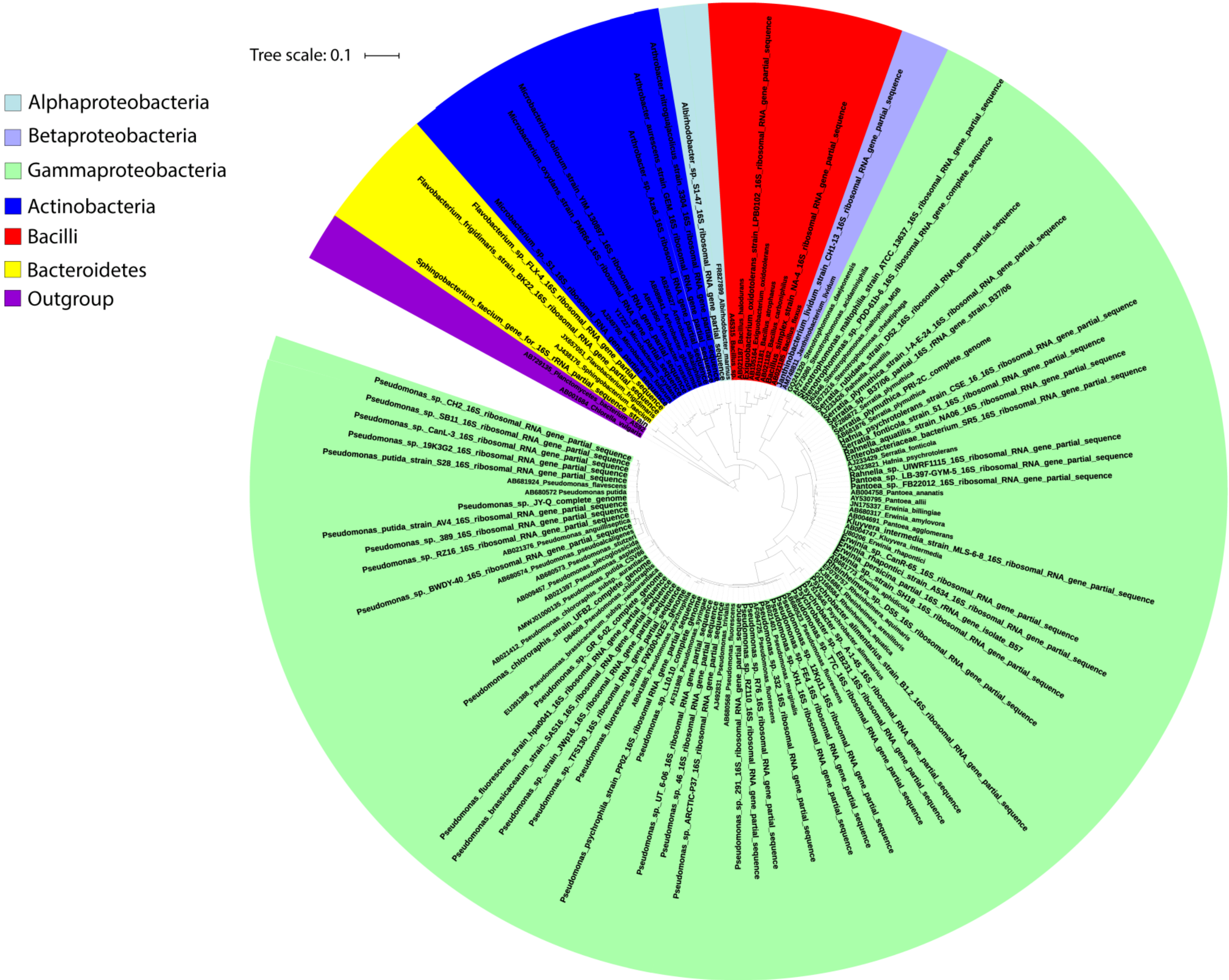

Figure S3 Phylogenetic tree of root-associated bacterial isolates based on Maximum Likelihood [ML] method. Navy, sky-blue, red, yellow, purple, and green color represent Actinobacteria, Alphaproteobacteria, Bacilli, Bacteroidetes, Betaproteobacteria and Gammaproteobacteria, respectively. Strains with long description are the bacterial isolates from this study, while strains with short name and accession number are the type strains obtained from SILVA rRNA database project.

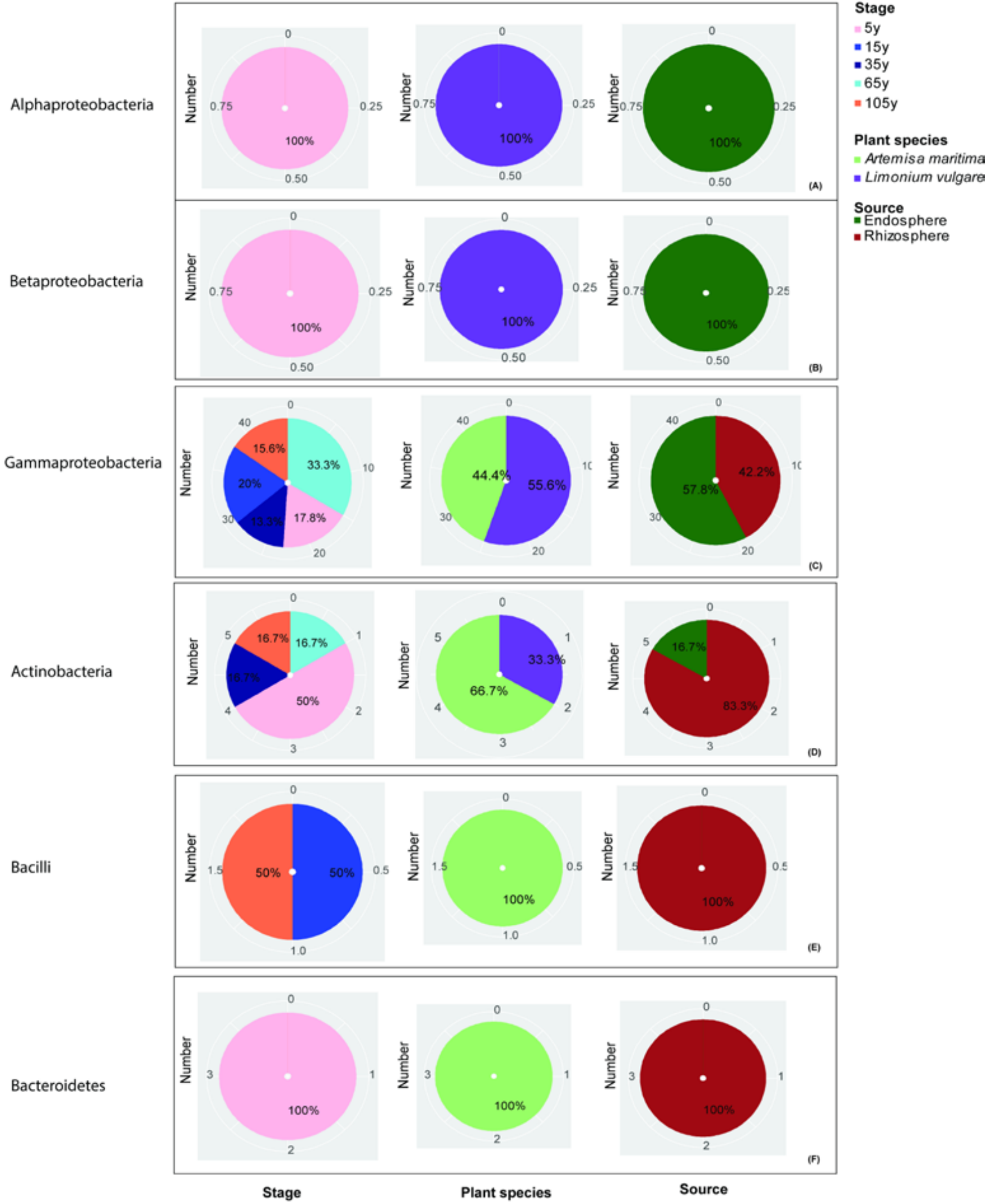

Figure S4 Distribution of isolates belonging to individual phyla along the successional stages, plant compartments and plant species. For successional stages, pink, blue, navy, cyan and orange color represent 5, 15, 35, 65 and 105-year stage, respectively. For plant compartments, red refer to rhizosphere isolates whereas green represent those obtained from the endosphere. Regarding plant species, the purple refers to *L. vulgare* isolates and green to *A. maritima*.

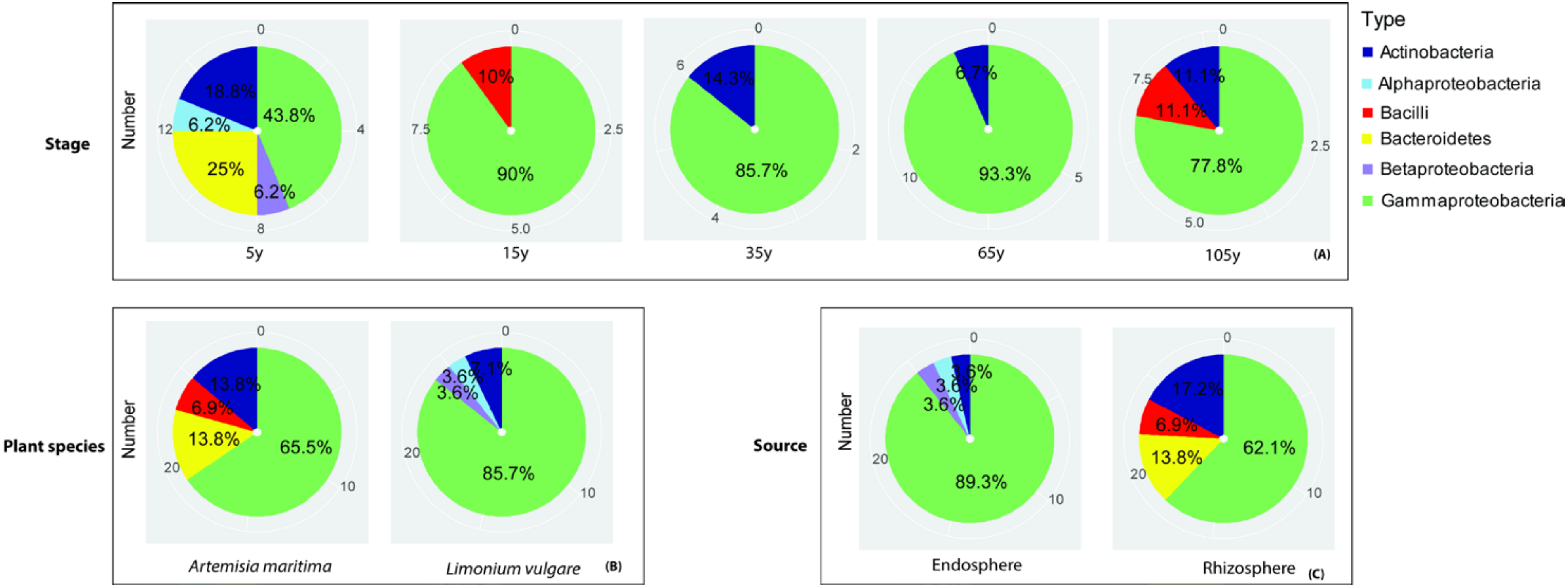

Figure S5 Phyla distribution in different treatments including successional stages, plant compartments and plant species. Navy, sky-blue, red, yellow, purple, and green color represent Actinobacteria, Alphaproteobacteria, Bacilli, Bacteroidetes, Betaproteobacteria and Gammaproteobacteria, respectively. The size of each sector represents the proportion of each treatment.

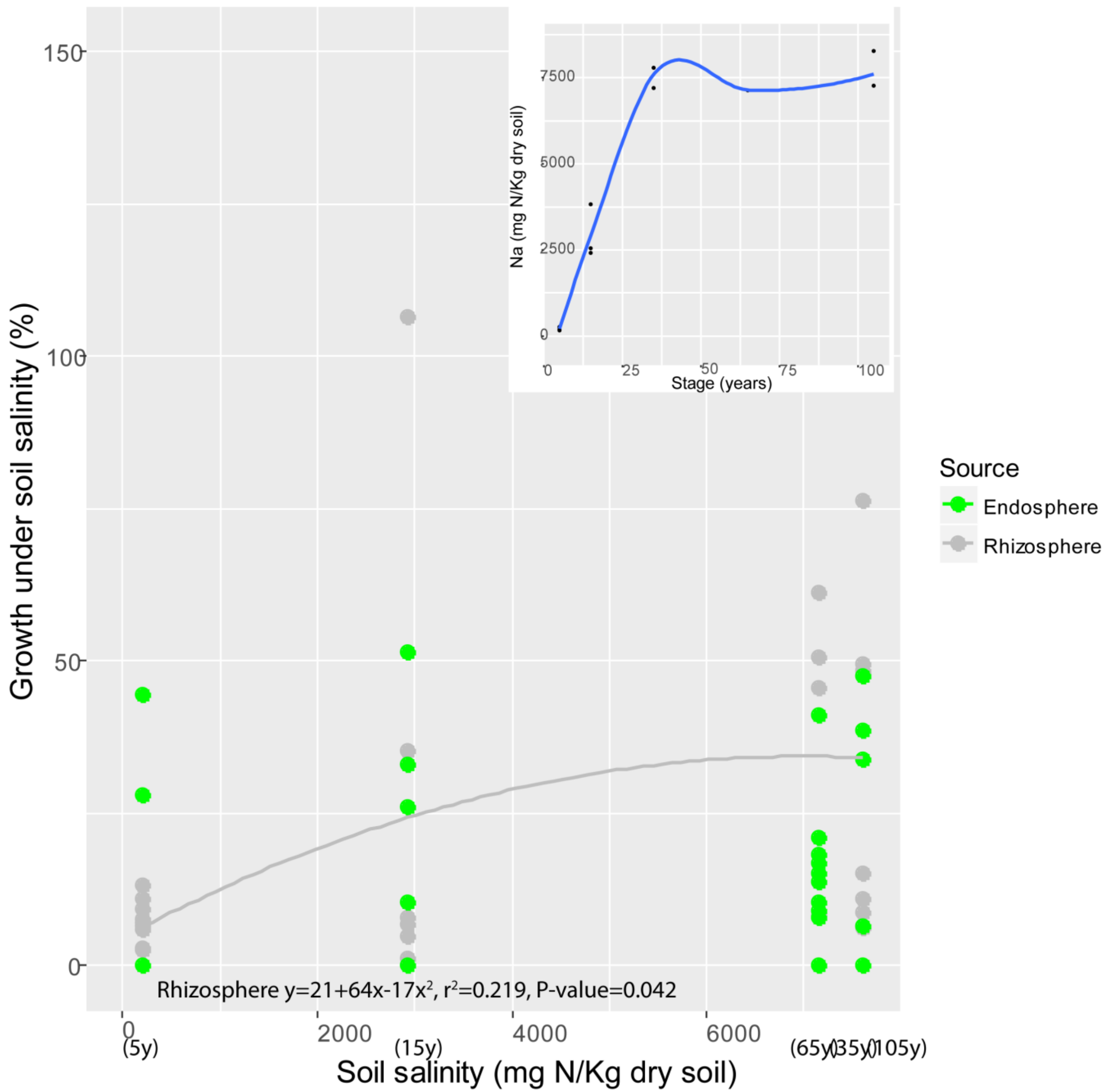

Figure S6 Consistent increasing salinity stress resistance of root-associated bacterial isolates corresponding to soil salinity changes along the succession. For plant compartments, grey refer to rhizosphere isolates whereas green represent those obtained from the endosphere.

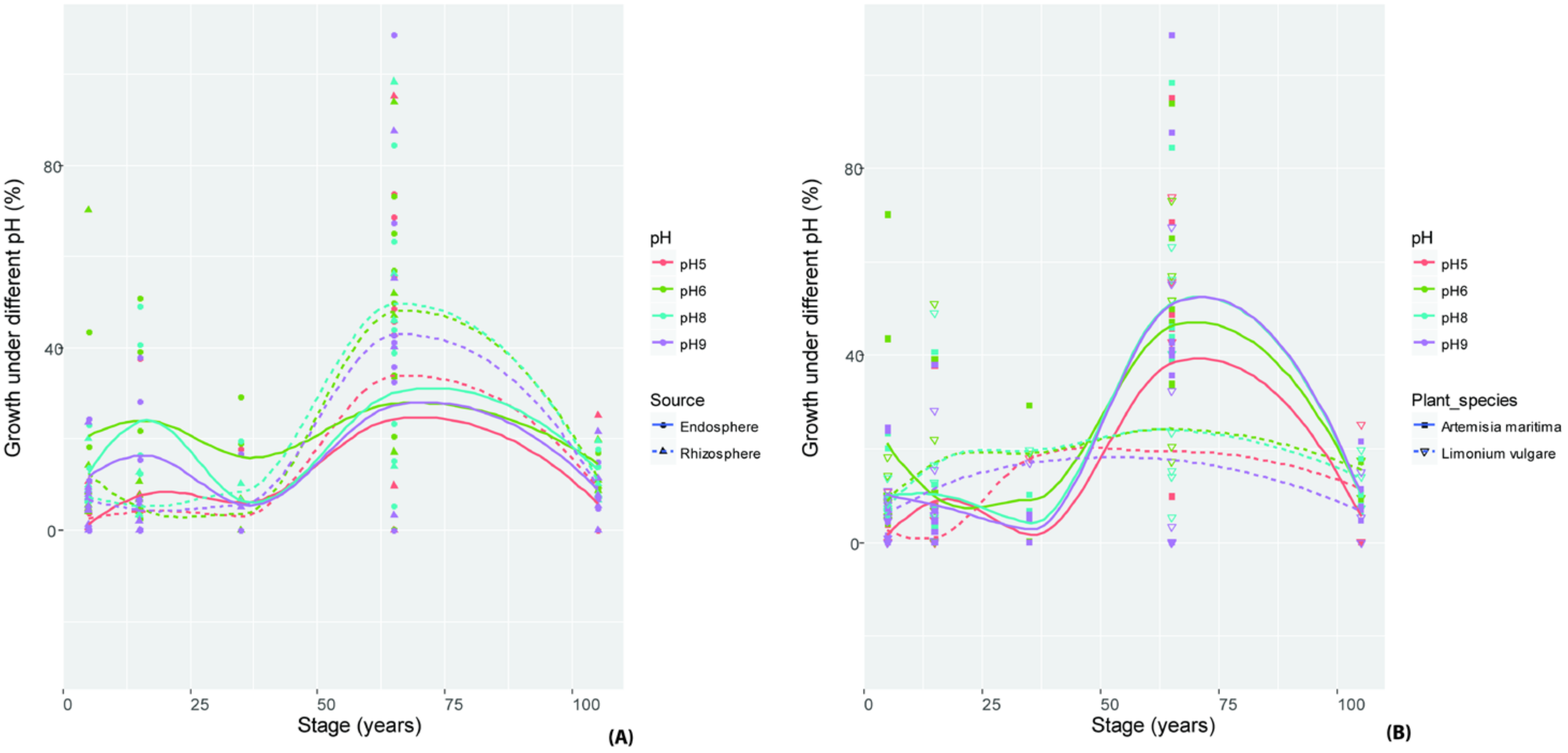

Figure S7 Variation of bacterial growth under different pH along the succession. (A) Comparison between plant compartments. Solid and dashed lines refer to endosphere and rhizosphere, respectively. (B) Comparison between plant species. Solid and dashed lines refer to *A. maritima* and *L. vulgare*, respectively.

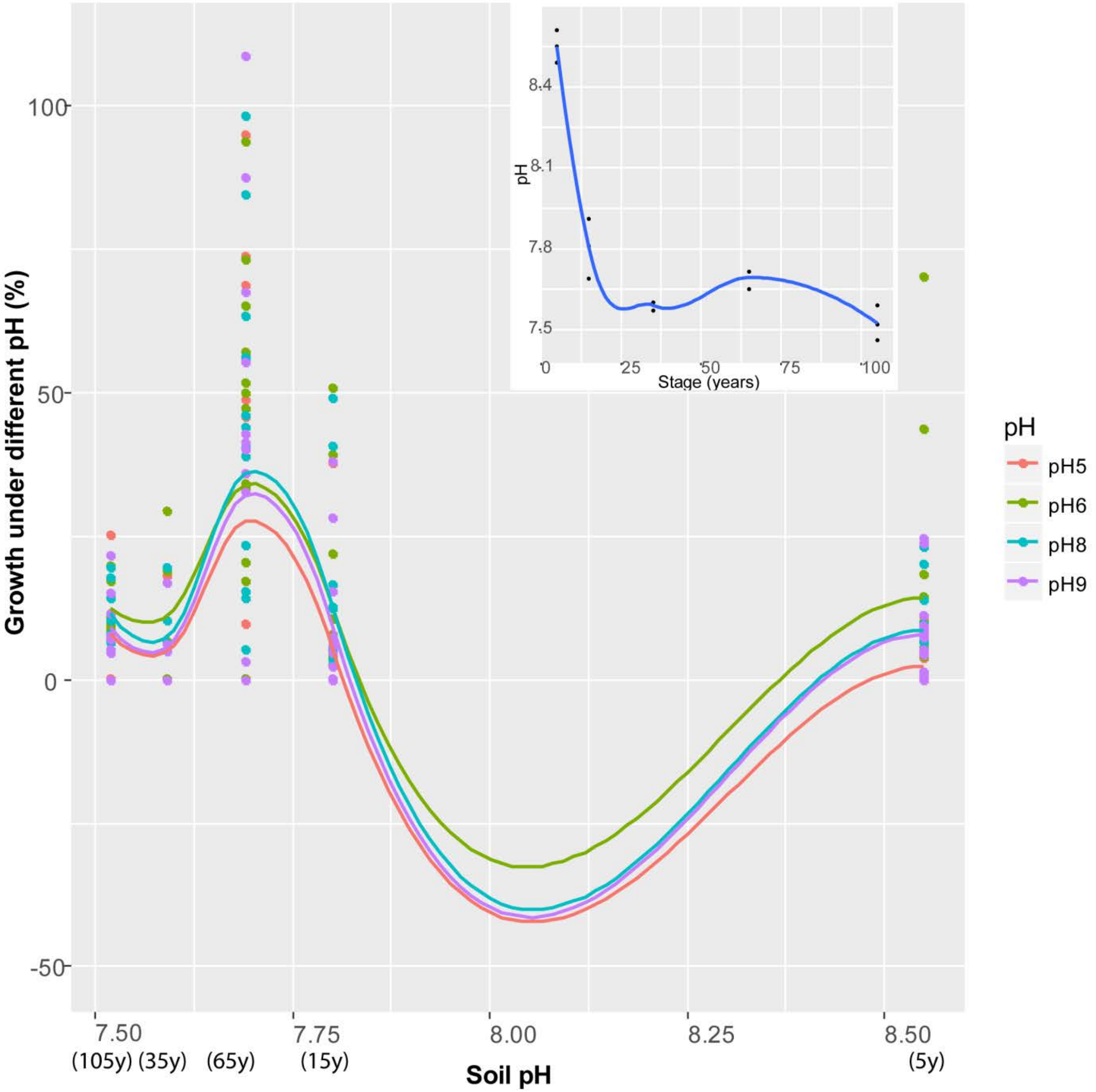

Figure S8 Variation of bacterial growth under different pH along the soil pH changes following the succession. Red, green, blue and purple color refer to pH levels of 5, 6, 8, and 9.
